# The Polish Society of Gynecological Oncology Guidelines for the Diagnosis and Treatment of Cervical Cancer (v2024.0)

**DOI:** 10.3390/jcm13154351

**Published:** 2024-07-25

**Authors:** Jacek J. Sznurkowski, Lubomir Bodnar, Łukasz Szylberg, Agnieszka Zołciak-Siwinska, Anna Dańska-Bidzińska, Dagmara Klasa-Mazurkiewicz, Agnieszka Rychlik, Artur Kowalik, Joanna Streb, Mariusz Bidziński, Włodzimierz Sawicki

**Affiliations:** 1 Profesor Sznurkowski Podmiot Leczniczy, ul. Stefana Żeromskiego 23A, 81-246 Gdynia, Poland; 2 Faculty of Medical Sciences and Health Sciences, University of Siedlce, 08-110 Siedlce, Poland; lubomirbodnar.lb@gmail.com; 3 Department of Clinical Oncology and Radiotherapy, Siedlce Cancer Center, 08-110 Siedlce, Poland; 4 Department of Tumor Pathology and Pathomorphology, Oncology Centre Prof. Franciszek Łukaszczyk Memorial Hospital, 85-796 Bydgoszcz, Poland; l.szylberg@co.bydgoszcz.pl; 5 Department of Obstetrics, Gynecology and Oncology, Collegium Medicum in Bydgoszcz, Nicolaus Copernicus University in Torun, 85-168 Bydgoszcz, Poland; 6 Department of Gynecological Oncology, Maria Sklodowska-Curie National Research Institute of Oncology, 02-781 Warsaw, Poland; agnieszka.zolciak@wp.pl (A.Z.-S.); agnieszka.rychlik@nio.gov.pl (A.R.); bidzinski.m@gmail.com (M.B.); 7 Department of Gynecologic Oncology, Second Clinic of Obstetrics and Gynecology, Medical University of Warsaw, 02-091 Warsaw, Poland; ad.bidzinska@gmail.com; 8 Department of Obstetrics and Gynecological Oncology, Medical University of Gdańsk, 80-210 Gdańsk, Poland; dklasa@gumed.edu.pl; 9 Department of Molecular Diagnostics, Holy Cross Cancer Center, 25-734 Kielce, Poland; artur.kowalik@onkol.kielce.pl; 10 Division of Medical Biology, Institute of Biology, Jan Kochanowski University, 25-406 Kielce, Poland; 11 Department of Oncology, Jagiellonian University Medical College, 31-008 Krakow, Poland; annaojs@interia.pl; 12 Department of Obstetrics and Gynecological Oncology, Medical University of Warsaw, 02-091 Warsaw, Poland; saw55@wp.pl

**Keywords:** cervical cancer, recommendation, diagnosis, imaging, surgery, radiotherapy, immunotherapy, early stage, locally advanced, metastatic, recurrent, persistent, adjuvant, follow up

## Abstract

**Background**: Recent publications underscore the need for updated recommendations addressing less radical surgery for <2 cm tumors, induction chemotherapy, or immunotherapy for locally advanced stages of cervical cancer, as well as for the systemic therapy for recurrent or metastatic cervical cancer. **Aim**: To summarize the current evidence for the diagnosis, treatment, and follow-up of cervical cancer and provide evidence-based clinical practice recommendations. **Methods**: Developed according to AGREE II standards, the guidelines classify scientific evidence based on the Agency for Health Technology Assessment and Tariff System criteria. Recommendations are graded by evidence strength and consensus level from the development group. **Key Results**: (1) Early-Stage Cancer: Stromal invasion and lymphovascular space involvement (LVSI) from pretreatment biopsy identify candidates for surgery, particularly for simple hysterectomy. (2) Surgical Approach: Minimally invasive surgery is not recommended, except for T1A, LVSI-negative tumors, due to a reduction in life expectancy. (3) Locally Advanced Cancer: concurrent chemoradiation (CCRT) followed by brachytherapy (BRT) is the cornerstone treatment. Low-risk patients (fewer than two metastatic nodes or FIGO IB2-II) may consider induction chemotherapy (ICT) followed by CCRT and BRT after 7 days. High-risk patients (two or more metastatic nodes or FIGO IIIA, IIIB, and IVA) benefit from pembrolizumab with CCRT and maintenance therapy. (4) Metastatic, Persistent, and Recurrent Cancer: A PD-L1 status from pretreatment biopsy identifies candidates for Pembrolizumab with available systemic treatment, while triplet therapy (Atezolizumab/Bevacizumab/chemotherapy) becomes a PD-L1-independent option. **Conclusions**: These evidence-based guidelines aim to improve clinical outcomes through precise treatment strategies based on individual risk factors, predictors, and disease stages.

## 1. Background

The Polish Society of Gynecological Oncology (PSGO) has developed the following recommendations for diagnosis, preoperative assessment for the surgical treatment, radiotherapy, systemic treatment, treatment of recurrent disease, and post-treatment surveillance of patients with cervical cancer, according to standards set by a guideline evaluation tool AGREE II (Appraisal of Guidelines for Research and Evaluation) [1].

Recommendations apply to women over the age of 18, who suffer from cervical cancer (they do not apply to patients with other malignant neoplasms of the cervix, e.g., neuroendocrine carcinomas, sarcomas, lymphomas, and melanomas).

The recommendations are intended for gynecologists, gynecological oncologists, surgeons, pathologists, geneticists, radiotherapists, clinical oncologists, general practitioners, palliative care specialists, and allied health care professionals.

The PSGO guidelines were developed using a six-step process:

1. Nomination of multidisciplinary development group (gynecological oncologist/pathologist/geneticist/clinical oncologist/radiation oncologist).
2. Identification of scientific evidence.
3. Formulation of guidelines.
4. Assessment of coherence with ESGO/NCCN guidelines.
5. Evaluation of guidelines by external reviewers.
6. Integration of external reviewers’ comments with original content of the guidelines.

The strength of scientific evidence was defined in agreement with the AOTMiT (the Agency for Health Technology Assessment and Tariff System) guidelines for scientific evidence classification [2] (Table 1). 

The grades of recommendation were based on the strength of evidence and the level of the consensus of the PTGO development group as described in Table 2. 

Clinicians utilizing the PSGO guidelines are urged to conduct thorough patient assessments for personalized care decisions.

### 1.1. Classification of Cervical Cancer

#### 1.1.1. Histopathological

Histopathological–Types Described in Table 3 [3] [Strength of Evidence V].

#### 1.1.2. Molecular

An extensive molecular characterization of 228 primary cervical cancers, the largest comprehensive genomic study of cervical cancer to date, unveiled novel insights into the disease, revealing previously unidentified genetic mutations and alterations. For instance, genes like ERBB3, CASP8, HLA-A, SHKBP1, and TGFBR2 have been identified as significantly mutated genes (SMGs) in cervical cancer for the first time. Importantly, ERBB3 has been singled out as a potential therapeutic target, indicating immediate clinical relevance.

Furthermore, the study identifies amplifications and fusion events involving the BCAR4 gene, a phenomenon never before reported in cancer. This discovery suggests that the indirect targeting of BCAR4, possibly through drugs like lapatinib, could be a promising avenue for treatment.

The molecular classification also sheds light on the immunological aspects of cervical cancer, with the identification of amplifications in genes encoding well-known immunotherapy targets like CD274 and PDCD1LG2. This suggests the potential efficacy of immunotherapy in treating certain cervical cancer subtypes.

Moreover, the study reveals a subset of cervical cancers with molecular profiles resembling endometrial tumors. This discovery suggests the possibility of repurposing therapies targeting proteins like PTEN and potentially ARID1A for the treatment of this specific subtype.

Overall, while molecular classification currently does not impact the management of cervical cancer, it holds significant potential for the near future. It offers a rationale for developing tailored therapeutic strategies by identifying specific molecular targets and pathways. This approach could facilitate the design of clinical trials aimed at treating different subpopulations of cervical cancer patients with greater precision and efficacy [4] [Strength of evidence IVA].

## 2. Screening for Cervical Cancer

In this section, we aimed to address critical inquiries regarding the organization of the screening program for cervical cancer. These included determining the protective benefits of cervical screening, comparing conventional and liquid-based cytology, assessing the superiority of HPV testing over cytology, evaluating the efficacy of high-risk HPV infection tests (mRNA vs. DNA), examining co-testing effectiveness, establishing appropriate screening intervals, and assessing whether vaccination against CIN2+ reduces the necessity for screening. We believe that providing evidence-based medical data will facilitate future Health Technology Assessment (HTA) analyses aimed at effectively organizing cervical cancer screening.

It is important to note that the PSGO recommendations do not cover the management of abnormal cervical cytology or interventions/treatment for cervical intraepithelial neoplasia.

### 2.1. Protective Benefits of Screening

The available evidence supports the conclusion that cervical screening does offer protective benefits (odds ratio 0.35; 95% confidence interval 0.30, 0.41) and is associated with a reduction in the incidence of invasive cervical cancer (relative risk 0.56, 95% confidence interval 0.42, 0.75) and cervical cancer mortality (risk ratio 0.65, 95% confidence interval 0.47, 0.90) [4,5]. [Strength of evidence IA, strength of evidence IIIA].

### 2.2. Conventional or Liquid-Based Cytology

Conventional cytology and liquid-based cytology (LBC) have similar sensitivity for the detection of cervical dysplasia [6,7]. [Strength of evidence IA, strength of evidence IA].

LBC had slightly higher sensitivity and NPV for the detection of CIN2, but there was no significant difference between the two methods [6]. [Strength of evidence IA]. The addition of HPV testing (molecular assays that detect HPV infection) to liquid-based cytology improves the sensitivity for the detection of cervical dysplasia [7]. [Strength of evidence IA].

### 2.3. HPV Testing or Cytology

There is no direct advantage of HPV testing over cytology in screening for cervical cancer [8]. [Strength of evidence IA] However, it is important to acknowledge the limitations, namely, the considerable variability in testing methodologies and screening/management protocols observed across the studies analyzed. While HPV tests exhibit a high sensitivity in detecting cases of CIN2+ and CIN3+, they may also result in increased unnecessary referrals. Nevertheless, a negative HPV test offers greater reassurance compared to a negative cytological test, as the latter carries a higher risk of false negatives, potentially leading to delays in receiving timely treatment [8]. [Strength of evidence IA].

Screening strategies employing a single initial HPV-positive test followed by colposcopy demonstrate remarkable effectiveness. Relative sensitivities for detecting CIN3+ using HPV-testing-based strategies, where HPV positivity mandates colposcopy, range from 0.8 to 2.1 compared to cytology. It is important to note that while HPV testing is more sensitive than cytology, it does result in significantly higher rates of colposcopy [9] with its risks (aftereffects including: pain, bleeding, infection, failure to diagnose, inadequate sampling, cost to the patient—e.g., time off work and psychological impact) and cost to the Health Care system [10]. [Strength of evidence IA and IIA].

Extended follow-up of four randomized-controlled trials comparing HPV testing versus cytology in screening for cervical cancer provided significant data for two screening rounds. HPV-based screening proved more effective in preventing invasive cervical cancers compared to cytology. Interestingly, screening protocols did not impact HPV testing efficacy. Greater protection against invasive cervical cancer was observed in women aged 30–35 years. Screening every 5 years demonstrated the highest protection, surpassing cytology every 3 years. Authors recommend HPV-based screening starting at age 30, with 5-year intervals [11]. [Strength of evidence III D].

### 2.4. High-Risk DNA HPV or mRNA HPV Tests

It has been demonstrated that there is no distinct difference in the accuracy and effectiveness between high-risk DNA HPV and mRNA HPV tests [12]. [Strength of evidence IA].

A study comparing primary screening methods in a hypothetical cohort of 2.25 million women from UK screening studies found potential cost savings of GBP 15.4 million and averted 28,009 unnecessary colposcopies by using an mRNA assay instead of a DNA assay. This also resulted in 90,605 fewer unnecessary HR-HPV tests and 253,477 fewer cytology tests. Comparing data from other primary HPV screening trials showed consistent cost savings and reduced testing with mRNA assay. The study concludes that adopting an mRNA assay over a DNA assay would likely save costs and reduce unnecessary testing and procedures, benefiting both healthcare providers and women in the screening program [13]. [Strength of evidence IIID].

### 2.5. Co-Testing (Conventional Cytology)

Systemic review of eight randomized clinical trials (RCTs) and five cohort studies comparing primary hrHPV screening alone or hrHPV co-testing (both hrHPV testing and cytology) with cytology (Papanicolaou [Pap] test) screening alone revealed that primary hrHPV screening detected higher rates of CIN3+ at the first-round screening compared with cytology.

Co-testing trials did not show initial increased CIN3+ detection. Both hrHPV screening strategies had higher false-positive and colposcopy rates than cytology, which could lead to more treatments with potential harms [14]. [Strength of evidence IB].

### 2.6. Co-Testing (Liquid-Based Cytology)

An approach involving combined liquid-based cytology (LBC) and HPV testing, followed by colposcopy for women with moderate dyskaryosis or worse, as well as HPV-positive mild dyskaryosis/borderline changes, while returning women with negative cytology or HPV-negative mild dyskaryosis/borderline changes to routine recall, demonstrated that using HPV testing as the primary screening method was significantly more protective over a six-year follow-up period compared to the prevailing practice of relying solely on LBC [15]. [Strength of evidence IIB].

### 2.7. Screening Intervals

The available research indicates that the level of protection remains similar 10 years after a negative HPV test and 3 years after a negative cytology test [16]. [Strength of evidence IIIC]. These findings suggest that a considerably longer screening interval can be considered following a negative HPV test compared to a negative cytology test.

Around three-quarters of women with HPV infection and normal cytology clear their infections within approximately 3 years, with a low risk of CIN3+ (1.5%) during this period. Moreover, approximately 40% of women who remained HPV positive had cleared their initial infection and acquired a new HPV type. Women with type-specific persistent infections have about six times higher cumulative risks of CIN3+ compared to those with new infections.

Implementing triage strategies based on HPV persistence could help reduce unnecessary referrals for women with new (and mostly transient) infections. Moreover, HPV assays that can identify HPV types 31, 33, 45, 52, and 58 alongside 16 and 18 could offer significant value in both triage and primary HPV testing [15,16]. [Strength of evidence IIB and IIIC].

Supporting an extension of screening intervals, regardless of the test assay employed, to five years after a negative HPV test in women aged 25–49 years, and even longer for those aged 50 years and older, as well as maintaining the screening interval at three years for HPV-positive women who have negative HPV tests upon early recall, is substantiated by findings from a large observational study [17]. [Strength of evidence IIIA].

### 2.8. Does Vaccination against CIN2+ Reduce the Need for Screening?

Vaccination against CIN2+ does appear to reduce the need for screening in certain age groups.

Women vaccinated at ages 16 years and younger or 17–19 years demonstrated significantly lower incidence ratios of cervical cancer compared to unvaccinated women. Specifically, for those vaccinated at ages 16 years and younger, the incidence ratio rate (IRR) was 0.14 (95% CI ¼ 0.04 to 0.53), while for those vaccinated at ages 17–19 years, the IRR was 0.32 (95% CI ¼ 0.08 to 1.28).

However, for women aged 20–30 years at the time of vaccination, the incidence rate was initially higher than among unvaccinated women (IRR ¼ 1.19, 95% CI ¼ 0.80 to 1.79). Yet, with increasing buffer periods, the incidence rate slightly decreased, indicating a potential benefit with time (IRR ¼ 0.85, 95% CI ¼ 0.55 to 1.32, with a 4-year buffer period) [18]. [Strength of evidence IIIA]. Moreover, another “real-world” study from Scandinavia reaffirmed the age-related effectiveness of vaccination in preventing cervical cancer. In this study, the incidence rate ratios were 0.12 (95% CI, 0.00 to 0.34) among women vaccinated before the age of 17 years, and 0.47 (95% CI, 0.27 to 0.75) among those vaccinated at the age of 17 to 30 years [19]. [Strength of evidence IIIA]. The above results are supported by another study showing lower positive predictive values (PPVs) for cytology and lower risk of developing CIN2+ in vaccinated women <20 years old. In conclusion, while vaccination against CIN2+ shows significant efficacy in reducing the risk of cervical cancer in women aged <16 years and <20 years, there is no discernible beneficial effect in reducing cancer risk for women vaccinated at ages > 20 years old [20]. [Strength of evidence IVA].

Hence, the necessity for screening may be diminished only in younger age groups (vaccinated < 20). Nonetheless, the persistence of both parameters—the incidence ratio rate and the positive predictive value (PPV) for CIN2+—at levels deemed too high underscores that primary prevention cannot entirely replace secondary prevention.

Additional crucial reasons for screening, irrespective of vaccination status and age, is that cervical cancer can arise from infections with HPV types not targeted by vaccinations [21] and could develop without HPV infection [22,23]. [Strength of evidence IIIA and IVA].

## 3. Diagnosis

### 3.1. Cervical Biopsy

The biopsy of the cervix is recommended for:

(1) Women with clinically visible tumors [best practice] [Strength of evidence V] (grade of recommendation 2B).

In such scenarios, it is recommended to perform a deep core biopsy of the tumor along with endocervical curettage, if feasible [24] [strength of evidence IVA] (grade of recommendation 2B). The preferred biopsy technique is the core needle biopsy (CNB), as it provides more reliable information for predicting future treatment modalities compared to superficial biopsy (e.g., lymphovascular space invasion (LVSI), stromal invasion (SI)) [24,25] [strength of evidence IVA, IVA] (grade of recommendation 2B).

For tumors < 2 cm, a deep core biopsy should be followed by conization to assess SI if not available from CNB specimen [26] [strength of evidence IVA] (grade of recommendation 2B).

(2) Women without evidence of cervical tumor but high-grade squamous intraepithelial lesion (HSIL) found during cytology screening [27] [strength of evidence IIIA] (grade of recommendation 2A).
(3) Women without evidence of cervical tumor but with atypical squamous cells—cannot exclude HSIL (ASC-H) or atypical cells of undetermined significance/ASC-US/found during cytology—having a positive test for oncogenic types of HPV [28,29] [strength of evidence IVB, IVA] (grade of recommendation 2B).
(4) Postmenopausal women without evidence of cervical tumor but with low-grade squamous intraepithelial lesion (LSIL) found during cytology, having a positive test for oncogenic types (including HPV 53) of HPV [30] [strength of evidence IVA] (grade of recommendation 2B).

The preferred biopsy method for cases without evidence of cervical tumor is a colposcopy, as demonstrated by its efficacy in detecting LSIL+ and HSIL+. When utilized to detect LSIL+, the colposcopy yielded a combined sensitivity of 0.92 (95% CI 0.88–0.95) and specificity of 0.51 (0.43–0.59). Similarly, for detecting HSIL+, the colposcopy displayed a combined sensitivity of 0.68 (0.58–0.76) and specificity of 0.93 (0.88–0.96) [27] [strength of evidence IIIA] (grade of recommendation 2A).

### 3.2. Clinical Significance of Pathological Features of the Biopsy Specimen

(1) Core biopsy of the macroscopic tumor along with endocervical curettage followed by conization for tumors smaller than 2 cm.

Objective: To verify the existence of cancer and furnish pathological features such as SI and LVSI. These factors are pivotal in determining the suitability of a patient with a tumor < 2 cm for a simple hysterectomy (see Section 4.2: Surgery—Evidence) or for those with tumors >2 cm <4 cm for either surgery or radiotherapy.

(2) Biopsy of the suspicious cervical lesions identified with or without colposcopy after abnormal cytology: HSIL, ASC-H, ASCUS HPV-positive, or menopausal LSIL HPV-positive.

Objective: To identify or exclude precancerous lesions such as HSIL or microscopic invasion (T1A1, T1A2) in specific areas of the cervix.

(3) Excisional biopsy of the cervix (cold knife conization) for histologically confirmed HSIL or T1A1 in cases lacking LVSI assessment after biopsy.

Objective: To exclude the presence of T1A or T1A2 lesions for HSIL, provide information on LVSI for T1A1/T1A2, and evaluate the margins of resection.

### 3.3. Clinical Significance of Pathological Features of the Post-Surgery Specimen

#### 3.3.1. Uterus

Objective: To evaluate surgical quality by examining the presence of residual disease in resection margins and provide data essential for calculating the risk of recurrence according to the Sedlis and Peatres criteria. This information is pivotal for determining the necessity of adjuvant radiotherapy.

#### 3.3.2. Nodes-Lymph Node Dissection /LND/

##### Number

As per the UICC guidelines, a pelvic lymphadenectomy specimen should contain six or more lymph nodes. However, if this count is not met and the resected lymph nodes are negative, the carcinoma should still be classified as pN0 [31] [strength of evidence V].

Apart from the arbitrary minimum number of nodes proposed by the UICC, there is no internationally accepted minimum for the number of resected lymph nodes required as part of a lymphadenectomy for cervical cancer.

##### Lymph Node Status

The most prognostic factor for cervical cancer is the presence of metastatic lymph nodes. Identification of metastatic nodes indicates patients at high risk of recurrence and serves as a predictor for adjuvant treatment, according to Peters criteria.

##### Lymph Node Ratio (LNR)

The ratio of positive to negative lymph nodes serves as a prognostic indicator in early-stage cervical cancer [32] [strength of evidence IIIE]. It has been identified as an independent predictor of both overall survival and disease-free survival in patients with squamous cell carcinoma (SCC) [33] [strength of evidence IIIE].

##### Nodes–Sentinel Lymph Node Biopsy SLNB

The role of ultrastaging in SLNB remains controversial (refer to the SURGERY section for evidence).

A comprehensive description of all essential clinical elements in the histopathological report is provided in Supplementary File S1.

### 3.4. HSIL Management

Excisional biopsy (conization) is recommended upon confirmation of HSIL via biopsy [best practice] [strength of evidence V] (grade of recommendation 2B). This procedure preserves lymph outflow, facilitating potential future sentinel lymph node biopsy if invasive cancer is detected within the cone [34,35] [strength of evidence IIA, IVA].

Conization aims to diagnose and treat by excluding cancer invasion.

The absence of invasion and negative margins may lead to the patient being considered cured [expert opinion] [strength of evidence V].

Patients initially diagnosed with HSIL on cervical biopsy, followed by negative cold knife conization specimens, exhibit a 6% recurrence rate for cervical intraepithelial neoplasia 2+ (CIN2+), while those with negative or CIN1 specimens have a 3.6% recurrence rate [36] [strength of evidence IA]. Therefore, intensified screening including high-risk mRNA HPV or at least DNA HPV testing is necessary for these high-risk patients [expert opinion] [strength of evidence V] (grade of recommendation 2B).

Studies show that vaccination during follow-up after conization reduces the recurrence rate of CIN [37] [strength of evidence IIA]. It is thus recommended to advise patients to undergo vaccination against CIN2+ after radical excision of HSIL during conization (grade of recommendation 2B).

Note: Avoid Sturmdorfa suture usage post-conization due to potential complications and hindrance in post-conization screening effectiveness [38] (expert opinion) [strength of evidence IVA, V] (grade of recommendation 2B).

Women with CIN2+ undergoing cervical conization via loop electrosurgical excision procedure (LEEP) have a 12% recurrence rate, twice that of cold knife conization, dependent on excised canal length [39] [strength of evidence IIID]. The positive margin rate after cold knife conization was significantly lower than after LEEP (5.8% vs. 12.09%, *p* < 0.001) [40] [strength of evidence IIID]

While LEEP is diagnostically accurate, its worse radicality, recurrence risk, and potential impact on lymph outflow make it less suitable for HSIL management (grade of recommendation 2A).

#### HSIL with Positive Margins after Conization

Studies indicate that the transformation of persistent disease into recurrent HSIL in women with positive margins occurs at a rate of 15–18%, which is higher compared to recurrence rate in women with negative margins (5.8–12%) [39,40] [strength of evidence IIID, IIID].

For those seeking fertility-sparing management, conization to attain negative margins followed by advice on vaccination against CIN2+ and a close follow-up is an option. Conversely, women opting for radical management should be offered a simple hysterectomy (minimally invasive techniques allowed—refer to the SURGERY section for evidence) (grade of recommendation 2A).

### 3.5. Management of T1A1, Negative LVSI, with Negative Margins Post-Conization

Systemic review of observational studies explored the link between LVSI and nodal metastases as well as survival in women with stage IA1 and IA2 cervical cancer. Results showed that less than 1% of stage IA1 patients without LVSI had positive nodes, contrasting with 7.8% of those with LVSI (*p* < 0.001). In stage IA2 cases, lymphatic metastases were found in 1.7% without LVSI compared to 14.6% with LVSI (*p* < 0.001). LVSI did not affect survival in IA1 cases but was associated with decreased survival in IA2 cervical cancer [41] [strength of evidence IIIA].

A small-scale retrospective analysis from a single institution focused on cases of IA1 without LVSI showed that conservative management following conization is both sufficient and safe. Notably, among 26 such cases, no recurrences were detected [42] [strength of evidence IVC].

A retrospective review of treatment approaches in 280 cases of stage IA1 and 44 cases of stage IA2 cervical cancer found that progression-free survival rates were comparable between patients treated with conization and hysterectomy for stage IA1 (92.3% and 98.8%, respectively; *p* = 0.07). The Cox regression analysis identified LVSI as an independent risk factor for recurrence in stage IA1 patients (OR, 12.14; *p* = 0.01). The study concluded that for stage IA1 patients with negative resection margins and no LVSI, conization may be an ideal treatment option, while for stage IA2 patients, a more conservative approach such as simple hysterectomy may be appropriate. LVSI was determined to be an independent risk factor for recurrence in stage IA1 cervical cancer [43] [strength of evidence IVC].

In another study assessing the prognosis and recurrence of microinvasive squamous cervical (MIC) cancer stage IA1 treated conservatively or by hysterectomy and followed up to 20 years, recurrences were observed in 7.3% (3/41) of cases in the conization group and 5.4% (5/92) in the hysterectomy group (*p* = 0.701). No significant differences were found in the risk of recurrence or overall disease-free survival time between the two treatment modalities.

Studies indicating a decrease in survival associated with minimally invasive surgery in cervical cancer patients do not include cases of IA1 LVSI negative (refer to the SURGERY section for evidence).

Recommendation: Hysterectomy (minimally invasion surgery (MIS) allowed should be considered as the definitive treatment option when fertility is not a concern. Regular long-term follow-up is essential for patients who undergo conization as the definitive treatment modality (grade of recommendation 2B).

**Figure 1:**
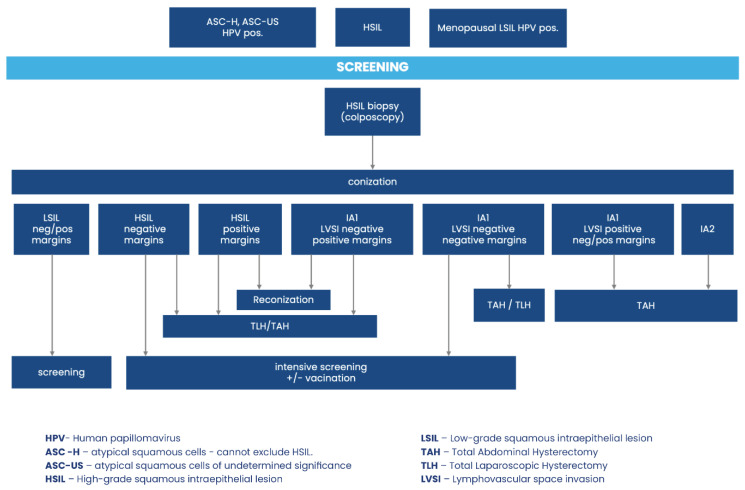
Illustration of the management of HSIL.

The management of HSIL identified in biopsy specimen is presented in Figure 1.

### 3.6. Imaging Prior to Treatment Decision

#### 3.6.1. Prior to Surgery

The best method of assessing the local advancement of cervical cancer (parametrial invasion [PMI], vaginal invasion, and bladder invasion) is magnetic resonance imaging (MRI) with contrast [44] (Strength of evidence IA).

Computed tomography (CT) is only useful in assessing the spread of cancer beyond the pelvis. The radiological assessment of the pelvis by CT is inferior to the MRI and expert ultrasound [44] (Strength of evidence IA).

Therefore, before treating cervical cancer, clinical and radiological staging should be performed based on gynecological examination, pelvic MRI, and the CT of the abdomen and chest (grade of recommendation 1). In cases where doubts persist regarding the extent of spread beyond the pelvis, a positron emission tomography–computed tomography (PET–CT) may be utilized [expert opinion] (grade of recommendation 2B).

Regarding the detection of lymph node metastases, recent metanalysis of 8 studies on CT, 38 studies on MRI, and 42 on PET–CT revealed that all of these modalities consistently have poor sensitivity (0.29–0.69) and high specificity (0.88–0.98). This is mainly because metastatic nodes are evaluated based on the size on the CT and MRI or the elevated radiotracer uptake on the PET—criteria that are well known to have limitations for detecting nodal micro-metastases in cervical cancer [45] [strength of evidence IIIA] and across several types of pelvic malignancies [46,47,48] [strength of evidence IIIA, IIIB, IIIB].

Thus, the surgical assessment of lymph nodes is still obligatory in early-stage tumor cases with radiologically unsuspicious nodes (SLNB for T1B1/2 or PLND + PALND for T1B3/4; both should be performed prior to hysterectomy) [49] [strength of evidence IIA]) (grade of recommendation 1).

#### 3.6.2. Prior to Chemoradiation

Extended para-aortic field radiotherapy, in addition to standard chemoradiation therapy, is recommended for patients with metastatic paraaortic lymph nodes (PALNs). However, such an extended radiation field from the pelvis to the upper abdomen significantly increases toxicity, such as radiation-induced enteritis [50,51] [strength of evidence IIIB, IIIB]. Therefore, an accurate determination of nodal status in advanced cervical cancer (any T, N1) significantly influences treatment burden and disease outcomes.

Meta-analysis of 18 cohorts in 16 studies demonstrates a significant rate of upstaging in patients with cervical cancer by laparoscopic PALN dissection after imaging (PET–CT or MRI) suggested no PALN metastases, particularly in patients with pelvic nodal metastases. The false-negative rate of PET–CT and MRI or CT imaging for the detection of PALN metastasis should be considered in clinical practice [52] [strength of evidence IA]

The elevated incidence of PALN metastases in patients with pelvic nodal metastasis suggests considering diagnostic PALN dissection for these cases rather than solely relying on imaging [52] [strength of evidence IA].

Therefore, the surgical staging of PALNs for the feasible patients with confirmed pelvic lymph node metastases, planed nor adjuvant (after hysterectomy) either primary chemoradiation, [strength of evidence IA] should be considered (grade of recommendation 1).

Clinico-radiological/cr/FIGO (2018) staging for cervical carcinoma is shown in Table 4 [53].

## 4. Treatment

### 4.1. General Rules–Common Misunderstandings in Nomenclature

The widely accepted classification of early cervical cancer and locally advanced cervical cancer (LACC), referring to clinico-radiological stages IA–IIA, originates from the historical division of cervical cancer into lower stages that are typically managed surgically, versus those requiring referral for radiotherapy, often delineated by the FIGO IIB cutoff. However, this classification deviates from the general oncological categorization of malignant solid tumors.

In standard oncology, “early cancer” refers to the confinement of the disease to the primary site, whereas “advanced” indicates tumor extension beyond the boundaries of the affected organ and/or involvement of regional lymph nodes. In the cases of cervical cancer, extension to the upper vagina and parametrium has been considered as involvement of the primary site (early cancer). This is because the cervix, upper vagina, and parametrium share the same Mullerian compartment and lymph node drainage patterns [54] [strength of evidence V]. In standard oncology, the involvement of the parametrium and/or vagina typically suggests a locally advanced stage of cancer rather than early-stage disease. “Disseminated cancer” signifies malignancy spreading to distant organs and/or non-regional lymph nodes.

To enhance the understanding of the proposed decision-making processes in cervical cancer management, these recommendations will utilize terms according to their established oncological definitions.

There are three clinical situations (radiologic/clinical rcTNM):

- Early and locally advanced cancer (early-cancer confined to the cervix, locally advanced cancer confined to parametrium and upper vagina rcFIGO IA/II);
- Advanced cancer (cancer extending to the pelvic side wall or adjacent organs rcFIGO IIIA-IVA);
- Disseminated cancer (rcFIGO IVB, any T any N, M+). Distant metastasis (including peritoneal spread; involvement of supraclavicular, mediastinal, or distant (inguino-femoral) lymph nodes; and lung, liver, or bone).

Only early and advanced cancers are treated with curative intent. The objective of such management is to eradicate the entire cancer tumor bed, encompassing both the cancerous lesion and the draining lymph nodes. Complete removal of the cancer with a margin of healthy tissue is essential to achieve the absence of residual disease (R0). Additionally, if necessary, the lymphatic drainage of the tumor should be inactivated to prevent distant dissemination. To achieve this goal, we can introduce surgery or radiotherapy.

Q: What is the preferred treatment modality for early and locally advanced cervical cancer: surgery or radiotherapy? (for clinic-radiological/cr/FIGO stage I/II)

A: There are no significant differences in disease-dependent survival time (OS) between surgery and radiotherapy for the treatment of early stage and locally advanced cervical cancer [55,56,57] [strength of evidence IIA, IVA, IVA].

However, there are studies supporting the choice of surgery (non-randomized–only observational based on SEER epidemiological database analyses), where the demonstrated advantage comes from the fact that surgery was significantly more often chosen in younger women with smaller tumors, etc.) [58,59] [strength of evidence IIIB, IIIB].

Q: So, what factors should influence the decision-making regarding the choice of method in the case of cr FIGO I-II?

A: The choice of method (surgery or radiotherapy) should be entirely individualized and depends on:

- Menopausal status (expected lifespan) [60] [strength of evidence IIa].
- Comorbidities (impact on operability) [60] [strength of evidence IIa].

For postmenopausal women with comorbidities, radical radiotherapy appears to be a safer option than surgery for early and locally advanced cervical cancer. It is recommended to choose the treatment modality with a lower risk of complications [expert opinion] [strength of evidence V] (grade of recommendation 2B).

- Histopathological type (poor response of adenocarcinoma /AC/ to radiotherapy [61] [strength of evidence IIID] and worse prognosis compared to the corresponding stage of SCC) [60,62] [strength of evidence IIIE, IIA];
- Parametrial involvement (cr FIGO IIB).

No randomized controlled trial (RCT) has specifically targeted stage IIB cervical cancer to compare surgery-based treatment with radiation therapy (RT)-based treatment. Several small retrospective studies have reported comparable overall survival (OS) rates between radical hysterectomy (RH), plus adjuvant radiotherapy (RT), and primary concurrent chemoradiotherapy (CCRT) in patients with stage IIB cervical cancer (RH 78% vs. CCRT 77%, *p* = 0.97) [56,57] [strength of evidence IVA, IVA]. However, most patients (90.5–100%) required adjuvant therapy due to the presence of unfavorable prognostic factors. For instance, the incidence of pelvic node metastases increased threefold from 13.3% to 37.8% when the parametrium was involved [63] [strength of evidence IVA].

Radical surgery combined with chemoradiation has more risks of side effects and long-term health problems compared to chemoradiation alone. These risks include issues like bowel obstruction, leg swelling, and chronic bladder problems [57] [strength of evidence IVA].

Based on the available data, surgery is not recommended in radiologically suggested parametrial involvement of cervical cancer (rc FIGO IIB) (grade of recommendation 2B)

- Tumor size

In 1990, a cut-off of 3 cm was proposed as an appropriate criterion for selecting between surgery and radiotherapy as primary treatment, following the publication of the Gynecologic Oncology Group study [64] [strength of evidence IVA], which was confirmed by recent long-term follow-up data [60] [strength of evidence IIA].

The current data suggest a tumor size of ≤2.0 cm in the largest dimension as the cut-off point for stratification between surgery and radiotherapy in cervical cancer management. Patients with small tumors (≤2.0 cm) exhibited a significantly lower frequency of pelvic lymph node involvement compared to patients with tumor sizes ranging between 2.1 cm and 4.0 cm (13.3% vs. 23.4%; *p* = 0.001), with an odds ratio for pelvic lymph node involvement of 2.0 [95% CI: 1.0–3.8] [65] [strength of evidence IVA].

This finding was confirmed by another study, which demonstrated that FIGO stage I and IIA tumors with ≤2.0 cm in the largest dimension had significantly lower rates of lymph node involvement, whereas rates were doubled for tumor sizes ranging between 2.1 and 3 cm (11.5% vs. 23.6%; *p* < 0.001) [66] [strength of evidence IVA].

Additionally, it has been proven that surgically treated tumors > 4 cm in 82% of cases will require adjuvant radiotherapy due to a significantly reduced likelihood of achieving a negative margin and a higher incidence of metastatic lymph nodes [60] [strength of evidence IIA].

Considering all these data, it is estimated that 13% of surgically treated cr FIGO IB1, 23% FIGO IB2/IIA1, and 82% cr FIGO IB3/IIA2 cases will be postoperatively upstaged to FIGO IIIC or IIB and will require adjuvant radiotherapy.

It is important to note that surgery was confirmed as a better choice for patients with cervical adenocarcinoma regardless of the tumor diameter, since RT did not yield comparable survival results [60] [strength of evidence IIA].

Based on the above data, surgery is primarily recommended for SCC/AC tumors with a maximum dimension of ≤2.0 cm (operable cr FIGO IB1 cases). Consideration may be given to SCC/AC tumors between 2 and 4 cm (operable cr FIGO IB2 and IIA1)* and even larger than 4 cm for AC (operable cr FIGO IB3 and IIA2). However, for SCC tumors larger than 4 cm, surgery is not recommended (grade of recommendation 1).

*Sedlis criteria from core biopsy (if available) (see Section 4.3 Radiotherapy—evidence).

Surgery has been assessed for cr FIGO IB2-IIB, incorporating neoadjuvant chemotherapy to reduce tumor size for radical surgery. However, neoadjuvant chemotherapy before surgery (NACT-S) did not prove superior to concomitant chemoradiotherapy (CCRT) [67] [strength of evidence IIA].

### 4.2. Surgery—Evidence

#### 4.2.1. Uterus

Q: Which approach—open or minimally invasive surgery—is preferable for performing a hysterectomy in cases of operable cervical cancer?

Minimally invasive radical hysterectomy has been linked to lower rates of disease-free survival and overall survival compared to open abdominal radical hysterectomy in women with FIGO IA1 (LVSI positive)–IB1 cervical cancer [68] [strength of evidence IIA]. This finding was validated by a large epidemiological study involving 2461 women with FIGOIA2 or IB1, among whom 49.8% underwent minimally invasive surgery [69] [strength of evidence IIIE]. Systematic reviews and meta-analyses of observational studies further supported this conclusion, demonstrating that minimally invasive radical hysterectomy posed a higher risk of recurrence and death compared to open surgery in patients with early-stage cervical cancer (FIGO IA-IIA) [70] [strength of evidence IIIA].

Moreover, another systematic review and meta-analysis revealed that even patients with small cancer tumors (<2 cm, FIGO IB1) treated with minimally invasive radical hysterectomy had significantly worse outcomes compared to cases treated with laparotomy [71] [Strength of evidence IIIA].

The cause of inferior oncologic outcomes linked to minimally invasive surgery for early-stage cervical cancer remains elusive. It has been speculated that manipulation of lymph nodes with previously undetected low-volume disease could contribute to this phenomenon. However, the MILLAC study analyzed lymph nodes using pathologic ultrastaging in node-negative patients who experienced recurrence in the LACC (Laparoscopic Approach to Cervical Cancer) trial. The results showed no evidence of lymph node low-volume metastases among these patients initially. Therefore, it is improbable that the manipulation of lymph nodes containing clinically undetected metastases is the root cause of the heightened risk of local recurrence observed in the minimally invasive arm of the LACC trial [72] [strength of evidence IIIC].

Exploratory data from an observational study [73] [strength of evidence IVC] confirmed a poor prognosis for minimally invasive surgery (MIS). However, the data indicated comparable relapse rates between laparoscopically treated cases with protective colopotomy (7%, 3 out of 43) and those treated with open surgery (11%, 47 out of 402). Additionally, there was no discernible difference in overall survival between the 106 cases treated laparoscopically without the use of a uterine manipulator and the 402 cases treated with laparotomy. While these exploratory, small-size findings offer a potential pathway for selecting patients for minimally invasive surgery, further prospective randomized trials are necessary to validate this approach, especially given the high strength of evidence suggesting laparoscopy as a significant risk factor for death.

Significantly, randomized controlled trials [74,75] [strength of evidence IIA, IIA] have confirmed that there is no substantial difference in the quality of life and comparable rates of adverse events between minimally invasive and open radical hysterectomy for early cervical cancer (FIGO IA-IIA).

Based on the above data, it is not recommended to use minimally invasive surgery for the surgical treatment of FIGO IA-IIA cervical cancer (grade of recommendation 1).

Q: Which type of hysterectomy is recommended for cervical cancer surgery: simple or radical?

The description of hysterectomy types is provided in Supplementary File S2.

In a multicenter, randomized study of 700 women with early-stage, low-risk cervical cancer, simple hysterectomy/SH/proved non-inferior to radical hysterectomy (RH) for pelvic recurrence at 3 years, with fewer urologic complications. The trial compared both procedures, including lymph-node assessment, in patients with low-risk cervical cancer (lesions ≤2 cm with limited stromal invasion). The majority had stage IB1 tumors (91.7%), squamous-cell histology (61.7%), and grade 1 or 2 (59.3%). At a median follow-up of 4.5 years, pelvic recurrence rates at 3 years were 2.17% for RH and 2.52% for SH, with a minimal difference (90% CI −1.62 to 2.32). Urinary incontinence incidence was lower in the SH group both within 4 weeks (2.4% vs. 5.5%; *p* = 0.048) and beyond 4 weeks (4.7% vs. 11.0%; *p* = 0.003). Urinary retention rates were also lower for SH within 4 weeks (0.6% vs. 11.0%; *p* < 0.001) and beyond 4 weeks (0.6% vs. 9.9%; *p* < 0.001).

The authors cautioned against extending the study’s findings beyond patients meeting the low-risk disease criteria: lesions ≤ 2 cm with invasion < 50% of stromal tissue or depth < 10 mm [76] [strength of evidence IIA].

Consequently, SH is recommended specifically for tumors ≤ 2 cm with identified SI. Therefore, CNB (followed by conization if necessary) is suggested for such cases. If the extent of stromal invasion is unknown or uncertain, RH is recommended (grade of recommendation 1).

#### 4.2.2. Lymph Nodes (Staging)

Q: Is SLNB a reliable method for detecting lymph node metastases?

A systematic review of trials conducted before 2015 has confirmed the high sensitivity of SLNB in all early-stage cervical cancers (FIGO IB-IIA). The overall weighted detection rate was found to be 0.93 (95% CI 0.92–0.94), with a pooled sensitivity of 0.88 (95% CI 0.84–0.90). Subgroup analysis revealed varying sensitivity and detection rates depending on the tracer techniques and surgical methods used in conjunction with SLN procedures:

- Studies using combined techniques showed a sensitivity of 0.88 (95% CI 0.84–0.91) and a detection rate of 0.97 (95% CI 0.96–0.98).
- Studies using metastable technetium-99 reported a sensitivity of 0.87 (95% CI 0.78–0.93) and a detection rate of 0.90 (95% CI 0.87–0.93).
- Studies using blue dye indicated a sensitivity of 0.87 (95% CI 0.79–0.93) and a detection rate of 0.87 (95% CI 0.84–0.90).
- Studies employing laparotomy showed a sensitivity of 0.86 (95% CI 0.80–0.90) and a detection rate of 0.87 (95% CI 0.83–0.91).
- Studies utilizing laparoscopy demonstrated a sensitivity of 0.90 (95% CI 0.86–0.94) and a detection rate of 0.93 (95% CI 0.90–0.96).
- Studies employing robot-assisted surgery reported a sensitivity of 0.84 (95% CI 0.72–0.92) and a detection rate of 0.92 (95% CI 0.88–0.95).

The study concluded that SLNB performs well diagnostically for the assessment of nodal metastases in patients with FIGO IA-IIA cervical cancer [77] [strength of evidence IA].

Another systematic review of trials available up to 2015 indicated the highest reliability of SLNB for tumors smaller or equal to 2 cm (FIGO IB1), with an accuracy of 93% (range 88.8–96.5). However, for larger tumors (>FIGO IB1), the accuracy was significantly lower at 65.9% (range 59–72.3) [78] [strength of evidence IIIA].

This observation was further supported by a prospective multicenter study (SENTICOL1) assessing the sensitivity and negative predictive value (NPV) of SLN mapping in a cohort of 139 patients with cervical carcinoma ranging from FIGO IA1 LVSI positive to stage IB. The study showed that for tumors not exceeding 2 cm, combined labeling (technetium 99 and patent blue injection) for node mapping is associated with high rates of SLN detection (98%), high sensitivity (92%), and NPV for metastasis detection (98.2%). No false-negative results were observed in cases where SLNs were identified bilaterally [34] [strength of evidence IIA].

Q: Is a sentinel lymph node biopsy an oncologically safe procedure?

The Senticol 2 trial confirmed the results of the Senticol 1 study and supported the sentinel lymph node (SLN) technique as a safe technique for use in patients with early-stage cervical cancer (87.4% IB1) treated with SLNB only. Disease-free survival after 4 years was similar in patients treated with SLN biopsy and patients who underwent SLN biopsy followed by LND [79] [strength of evidence IIA].

The safety was also validated by an international, multicenter, prospective single-arm study focusing on SLNB without systematic pelvic lymphadenectomy (PLND) in patients with a broader range of tumor sizes: T1a1 L1–T1b2 (<4 or ≤2 cm for fertility sparing) and no suspicious lymph nodes on preoperative imaging. The surgical approach involved SLNB followed by hysterectomy or trachelectomy, with all excised SLNs undergoing comprehensive pathologic ultrastaging centrally evaluated for quality. In the case of metastatic SLN involvement, adjuvant chemoradiotherapy was advised, though the patient remained in the ITT cohort. The study revealed that the recurrence rate post-SLN biopsy with pathologic ultrastaging (6.06%) was similar to the reference recurrence rate of 7% reported in patients undergoing pelvic lymphadenectomy [80] [strength of evidence IIIC]. However, it is imperative to note that this was a single-arm study, with the treatment arm compared to a reference recurrence rate of 7% derived from other studies. The 6% recurrence rate reported in the SENTIX trial warrants careful interpretation, as the study excluded cases of fertility-sparing recurrences and included patients with metastatic SLNs who received chemoradiation as a preventive measure. The recurrence rate in the cohort with negative SLNB, where patients are simply observed, is indicative of oncological safety and appears to be higher than 6%.

The impact of SLNB alone versus PLND on survival for patients with early-stage cervical cancer was recently assessed in systemic review and metanalysis of observational studies. The results revealed that both 5-year disease-free survival and overall survival rate after SLNB alone are higher than 90% and do not differ from PLND survival data. Interestingly ultrastaging did not impact survival [81] [strength of evidence IIIA].

Q: What to choose: SLNB or LND?

##### Impact on Morbidity

Data from another study of the SENTICOL group has shown that lymphatic morbidity was notably lower in the SLNB arm compared to the SLNB + PLND arm, along with a reduced rate of postoperative neurological symptoms. However, there was no significant contrast in the occurrence of significant lymphedema between the two groups. Over a postoperative period of 6 months, the disparity in morbidity decreased. The study concludes that SLNB is linked to early reduction in lymphatic morbidity compared to PLND in early-stage cervical cancer [82] [strength of evidence IIA].

##### Impact on Therapeutic Sequence

Findings from the ABRAX study indicate that the completion of radical hysterectomy does not enhance survival in patients with intraoperatively detected lymph node involvement, irrespective of tumor size or histological type. If lymph node involvement is confirmed intraoperatively, hysterectomy should be avoided, and the patient should be referred for definitive chemoradiation [49] [strength of evidence IIIA].

Based on the findings of the ABRAX study, integrating SLNB with intraoperative pathologic ultrastaging into all scheduled hysterectomies can offer enhanced protection for patients by minimizing unnecessary surgeries compared to upfront LND with a pathological assessment of macroscopically suspicious nodes. This approach reduces treatment-related morbidity and facilitates the prompt initiation of chemoradiation therapy.

The procedure for performing SLNB is outlined in Supplementary File S3.

Recommendation:

SLNB is the preferred method for assessing lymph node status in tumors ≤ 2 cm, and it may be considered with caution for tumors >2 cm–<4 cm [grade of recommendation 2A].

If SLNB cannot be performed due to technical or logistical limitations (institutional resources), up-front LND with mandatory intraoperative histopathological assessment of suspicious nodes (if present) before hysterectomy remains a valuable alternative option in early-stage cases [grade of recommendation 2A].

If metastatic lymph nodes are confirmed intraoperatively, hysterectomy is not recommended [grade of recommendation 2A].

#### 4.2.3. Ovaries

Q: Is ovarian transposition (OT) safe and beneficial for young cervical cancer patients?

A systematic review and meta-analysis confirmed the effectiveness and safety of OT in cervical cancer patients undergoing radio-surgical treatment. OT maintains ovarian function with minimal risk of metastases to the transposed ovaries, despite an increased incidence of ovarian cysts. The study concluded that ovarian transposition offers a significant preservation of ovarian function [83] [strength of evidence IA].

Recommendation:

OT should be considered in younger cervical cancer patients regardless of the treatment modality (surgery and/or radiotherapy) (grade of recommendation 1).

**Figure 2:**
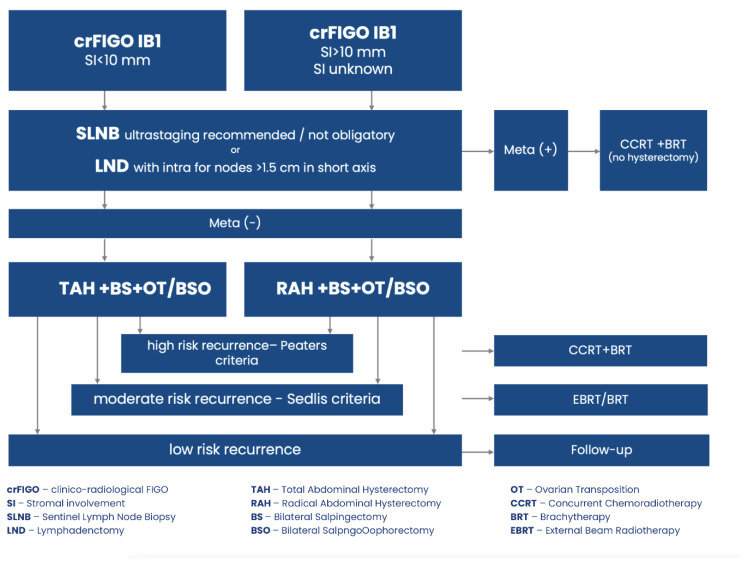
Illustration of the decision tree for treatment of cr FIGO stage IB1.

Treatment management of patients with clinicoradiological FIGO stage IB1 describes Figure 2.

**Figure 3:**
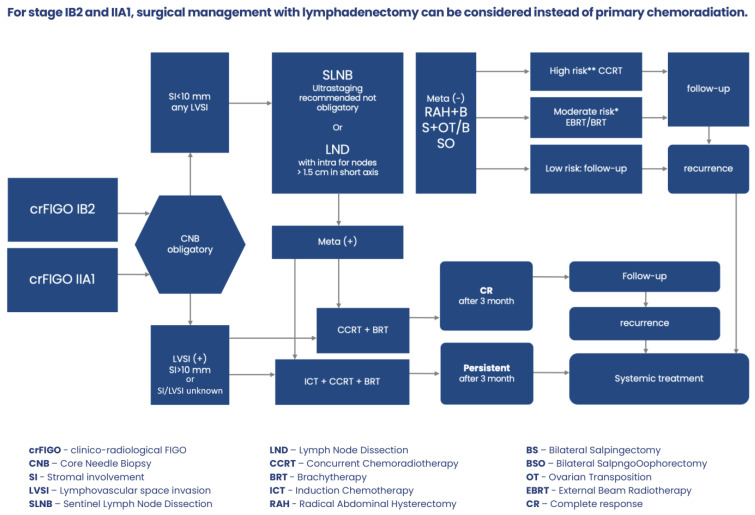
Selection criteria for surgical treatment of cr FIGO stage IB2 and IIA1. * Peters criteria, ** Sedlis criteria.

Treatment management of patients with clinicoradiological FIGO stage IB2-IIA1 describes Figure 3.

Q: Is salvage surgery (hysterectomy/pelvic exenteration) beneficial in cases of failed radical concurrent chemoradiation advanced cervical cancer, particularly in cases of crFIGO III/IVA?

Patients undergoing salvage surgery due to suspected residual disease on radiology showed residual disease on the salvage surgery specimen in 27–48% of cases [84] [strength of evidence IIIE].

However, patients with radiologically suspected or histologically confirmed residual disease after definitive chemoradiation (diagnosed with post-treatment radiological workup or biopsy) and treated with salvage hysterectomy (extrafascial or radical) and pelvic exenteration (anterior, posterior, or total) had a 32% recurrence rate, 40% mortality, and median OS of 32 months (range 9–239) during 38 months of follow-up [85] [strength of evidence IIIC].

Recommendation: The decision to perform salvage surgery must be approached with caution and should only be considered when persistent or recurrent disease is histologically proven (grade of recommendation 2A).

If the triplet regimen (chemotherapy + bevacizumab + atezolizumab) is available and feasible, systemic treatment stands as the optimal choice for persistent disease. 

### 4.3. Radiotherapy—Evidence

#### 4.3.1. Adjuvant Treatment after Surgery

Q: Why do we use adjuvant treatment after surgery for early-stage cervical cancer?

Incorrect patient selection or inadequate surgical technique can result in postoperative upstaging or residual disease at resection margins, respectively.

This occurs when preoperatively diagnosed early-stage cervical cancer is found to be advanced, spreading to the parametrium or lymph nodes. For instance, preoperative cr FIGO IA–T2A may ultimately become FIGO IIB or FIGO IIIC, respectively. Additionally, planned R-0 may ultimately turn out to be R1 (microscopic residual disease), with the vagina being the most common site of residual disease.

Exploratory data from randomized controlled trial (RCT) show that this subgroup of patients faces a high risk of recurrence (approximately 40%) and mortality (around 50%) following surgery alone. However, concurrent cisplatin-based chemotherapy with radiotherapy has emerged as the optimal adjuvant treatment option, significantly improving progression-free and overall survival [86] [strength of evidence IIA]. Consequently, criteria such as involved surgical margins, parametrial invasion, and lymph node metastases (Peters criteria) are now utilized to identify patients at high risk of recurrence for selection for adjuvant concurrent chemoradiation.

The GOG 92 study by Sedlis et al. [87] [strength of evidence IIA] categorized patients based on Sedlis criteria for adjuvant whole pelvic radiation or observation alone. It demonstrated that radiation therapy significantly reduced the recurrence rate to 15% compared to 28% in patients who received no further treatment, although overall survival did not show a significant improvement [HR 0.70, 90% CI 0.45–1.05, *p* = 0.074].

The Sedlis criteria pinpointed cases with a 30% risk of recurrence, labeling them as intermediate-risk patients, based on the scoring system outlined below:

- Presence of LVSI plus deep (outer third) cervical stromal invasion and tumor of any size;
- Presence of LVSI plus middle (one-third) stromal invasion and tumor size ≥ 2 cm;
- Presence of LVSI plus superficial (inner third) stromal invasion and tumor size ≥ 5 cm;
- No LVSI but deep or middle cervical stromal invasion and tumor size ≥ 4 cm

Recent research corroborates that pelvic radiotherapy following radical surgery notably diminishes the risk of recurrence and improves progression-free survival (PFS) among women diagnosed with Stage IB cervical cancer and exhibiting poor prognostic factors (according to the Sedlis criteria) [87,88] [level of evidence: IIA, IIA].

Recommendations: Surgically treated, cervical cancer patients deemed to have a moderate risk of recurrence based on the Sedlis criteria should undergo adjuvant pelvic radiotherapy (grade of recommendation 1). Conversely, patients identified as high risk for recurrence according to the Peters criteria following surgery should receive concurrent chemoradiation (grade of recommendation 1).

##### STARS Trial

Chinese patients with early-stage cervical cancer (crFIGOIB1-IIA2), exhibiting post-surgery risk factors, such as lymph node metastasis, positive parametrium or margins, lymphatic vascular space involvement, or deep stromal invasion, were randomly assigned to receive adjuvant radiation alone (RT), concurrent chemoradiation (CCRT), or sequential chemoradiation (SCRT) after radical hysterectomy and pelvic lymphadenectomy. The study demonstrated that compared to RT or CCRT, SCRT improved disease-free survival (DFS), reduced distant recurrence, and decreased the risk of death. These findings suggest that SCRT should be considered a preferable adjuvant treatment for these patients [89] [strength of evidence IIA].

While the results of the STARS trial are promising, several important questions remain unanswered. It is crucial to investigate the impact of pharmaco-genomics on therapy, particularly whether treatment effectiveness is limited by adverse effects influenced by patient characteristics, including ethnicity. Additionally, the reasons behind the improved outcomes in the sequential arm, such as the addition of taxane, timing of chemotherapy and radiation, or differences in dose intensity, need further exploration. Another potential factor to consider is the inclusion of patients who received neoadjuvant treatment, and their effects on treatment discontinuation, toxicity, and efficacy have yet to be fully elucidated.

Recommendation:

Before integrating it into the standard of care, we suggest awaiting the outcomes of other studies examining the use of chemotherapy post-chemoradiation, such as RTOG-0724 (NCT00980954), particularly in high-risk early-stage cervical cancer patients treated with radical hysterectomy.

In cases where resources are a factor, sequential chemoradiation (SCRT) following radical hysterectomy and pelvic lymphadenectomy might be worth considering, especially if there are extended waiting times for radiotherapy alone or concurrent chemoradiation (grade of recommendation 1).

Detailed data on radiation therapy and the concurrent chemoradiation sequential chemoradiation are described in Supplementary File S4.

#### 4.3.2. Curative Radical Radiotherapy-cr FIGO IB2-IVA Cases with Preserved Uterus

Multiple RCTs and systematic reviews have consistently demonstrated that CCRT followed by brachytherapy/BRT/significantly prolongs OS. As a result, CCRT has emerged as the standard of care for treating advanced stages of cervical cancer [90,91,92] [Strength of evidence IIA, IA, IIA].

The INTERLACE trial (NCT 01566240) demonstrated a significant enhancement in OS and progression-free survival/PFS/with upfront induction chemotherapy (ICT). Participants were randomized to receive ICT with weekly paclitaxel 80 mg/m^2^ and carboplatin area under the curve of 2 for 6 weeks followed by CCRT + BRT versus CCRT + BRT alone. The experimental arm showed a 9% improvement in PFS and an 8% improvement in OS at 5 years [93] [Strength of evidence IIA], marking the first time in over two decades since an OS benefit was reported in locally advanced cervical cancer treatment.

It is essential to consider the study population (70% UK) and note that a significant proportion of patients (77%) were staged II and (58%) had node-negative disease. Additionally, the median interval between ICT and CCRT was 7 days.

The CALLA trial (NCT 03830866) investigated the addition of immune checkpoint inhibitors (ICIs) to CCRT. Patients were randomized to receive the programmed death-ligand 1 (PD-L1) inhibitor, durvalumab, in combination with CCRT + BRT followed by a maintenance phase versus CCRT + BRT alone [CALA]. Although there was no statistically significant improvement in PFS in patients receiving durvalumab and CCRT + BRT compared to those receiving CCRT + BRT alone (65.9% vs. 62.1% at 24 months, respectively), the safety profiles were comparable between the two groups [94] [Strength of evidence IIA].

In contrast, the KEYNOTE-A18 trial (NCT 04221945) evaluated the PD-1 inhibitor pembrolizumab with CCRT + BRT and as part of maintenance therapy compared to CCRT + BRT alone. The primary endpoint, PFS, was met with a PFS of 67.8% versus 57.3% at 24 months in the CCRT plus pembrolizumab arm versus CCRT alone, respectively (hazard ratio [HR] = 0.70; 95% CI = 0.55–0.89) [95] [Strength of evidence IIA]. Moreover, the improvement in PFS with the addition of pembrolizumab to CCRT in patients with high-risk locally advanced cervical cancer was accompanied by QoL changes similar to those in the placebo + CCRT group. These results support the positive benefit–risk profile of this combination [96]

Ref. [96] Conference Paper (abstract 2850 in *International Journal of Gynecological Cancer*, March 2024, DOI: 10.1136/ijgc-2024-ESGO.53).

The observed differences in PFS between KEYNOTE-A18 and CALLA are likely attributed to variations in the study populations and the specific ICI used. KEYNOTE-A18 required two or more involved lymph nodes 1.5 cm in short axis, while CALLA only required one or more nodes 1 cm in short axis. This suggests that the addition of ICIs to CCRT may be most beneficial for a higher-risk population, with blunted effects in lower-risk populations where CCRT alone may suffice.

Recommendation:

CCRT followed by BRT remains the cornerstone of treatment for advanced cervical cancer patients (cr FIGO IB2-IVA) (grade of recommendation 1).

In low-risk patients (with less than two metastatic nodes or cr FIGO IB2-II), considering ICT followed by CCRT + BRT after a 7-day interval could be an option. This treatment is both accessible and affordable, making it suitable for low-resource settings. (grade of recommendation 1).

In high-risk patients (with two or more metastatic nodes* or cr FIGO IIIA, IIIB, IVA regardless of nodes), pembrolizumab in combination with CCRT and maintenance therapy represents the optimal choice (grade of recommendation 1).

*Metastatic nodes must have at least 1.5 cm in short axis

**Figure 4:**
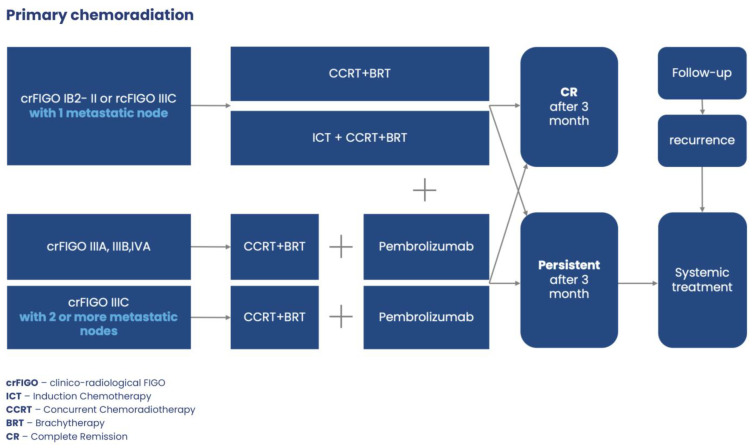
Illustration of the decision tree for managing cr FIGO stage IB2–IA1 (excluded from surgery) and FIGO IB3/IIA2–IVA.

The decision tree for managing of rc FIGO IB2-IA1 (excluded from surgery) and rcFIGO IB3-IIA1 Cases with Preserved Uterus) illustrates Figure 4. 

Detailed data on CCRT followed by BRT, up-front ICT to CCRT/INTERLACE/and ICIs in combination with CCRT and maintenance therapy/Keynote A18/are described in Supplementary File S5.

### 4.4. Systemic Treatment–Evidence

#### 4.4.1. First Line Treatment for Metastatic/cr FIGO IVB/Persistent* or Recurrent Disease

*The definitions of persistent and recurrent disease are provided in the follow-up section.

In the GOG-240 trial (NCT 00803062), the introduction of antiangiogenic therapy alongside conventional chemotherapy marked a significant stride in addressing the pressing clinical needs of recurrent or metastatic cervical cancer/rmCC/management. Prior to this advancement, the median OS stood at 13.3 months for this patient cohort. However, with the incorporation of bevacizumab into a platinum doublet regimen, the OS was extended to 16.8 months, demonstrating a notable improvement (HR = 0.77; 95% CI = 0.62–0.95). This landmark outcome established the combination therapy as the standard of care by 2014 [97,98] [strength of evidence IIA, IIA].

Concurrently, the Japan Clinical Oncology Group study (JCOG0505) ran alongside GOG-240, showcasing non-inferior outcomes in patients treated with carboplatin and paclitaxel compared to those treated with cisplatin and paclitaxel for stage IVB, persistent, or recurrent cervical cancer/rpmCC/[99] [strength of evidence IIA]. However, subgroup analysis revealed a discrepancy: patients who had not previously received cisplatin exhibited shorter OS with carboplatin plus paclitaxel compared to cisplatin plus paclitaxel (13.0 versus 23.2 months; HR = 1.571; 95% CI = 1.06–2.32) [99] [strength of evidence IIIA]. Consequently, this established carboplatin plus paclitaxel as the preferred platinum doublet for stage IVB or recurrent cervical cancer, except in cases where patients were cisplatin-naïve [99] [strength of evidence IIA].

The introduction of pembrolizumab for patients with rpmCC and a PD-L1-combined positive score (CPS*) of ≥1 alongside a platinum doublet (cisplatin or carboplatin plus paclitaxel), with or without bevacizumab, yielded significantly prolonged progression-free survival (PFS) and OS [100] [strength of evidence IIA]. The median PFS reached 10.4 months in the pembrolizumab group compared to 8.2 months in the placebo group (HR = 0.62; 95% CI = 0.5–0.77). Additionally, at the 24-month mark, the median OS was notably higher at 53% in the pembrolizumab arm versus 41.7% in the placebo arm (95% CI = 0.5–0.81; *p* < 0.001) [100] [strength of evidence IIA]. These findings were further reinforced by the final OS analysis across PD-L1 CPS ≥ 1 (HR = 0.60; 95% CI = 0.49–0.74), all-comer (HR = 0.63; 95% CI = 0.52–0.77), and CPS ≥ 10 populations [101] [strength of evidence IIA].

Notably, investigations into health-related quality of life among patients receiving pembrolizumab demonstrated no detrimental effects when added to chemotherapy with or without bevacizumab [102] [strength of evidence IIA], thus emphasizing the positive impact and value of pembrolizumab in the treatment of rpmCC.

**[CPS-score) The programmed death ligand 1 (PD-L1)-combined positive score was defined as the number of PD-L1-staining cells (tumor cells, lymphocytes, and macrophages) divided by the total number of viable tumor cells, multiplied by 100.

How to calculate CPS scores for PD-L1 is described in Supplementary File S1.

BEATcc (NCT 03556839) enrolled and randomized individuals with rmCC to treatment with cisplatin/paclitaxel and mandatory bevacizumab with or without the PDL1 inhibitor atezolizumab. The addition of atezolizumab resulted in significantly higher PFS and OS with a 38% reduction in the risk of progression and 32% reduction in the risk of death, respectively [103] [strength of evidence IIA]. Notably, the overall response rate (ORR) and duration of response were higher in the experimental arm versus placebo with an ORR of 84% versus 72% and a complete response rate of 32% vs. 20%, respectively [103] [strength of evidence IIA]. The toxicity profile was acceptable.

Triplet improved OS–San Diego 2024 [104] [strength of evidence IIA].

When comparing Keynote-826 to BEATcc, ORR was more pronounced in the BEATcc cohort (84%) compared to Keynote-826 (69%) [4,29,31]. The complete response was also more common in BEATcc (32%) versus Keynote-826 (26%). It is likely that these observed differences are a result of the uniform use of bevacizumab in BEATcc (100% compared to 63% in Keynote-826) and the synergistic effect that exists between platinum doublets, VEGF inhibitors, and ICIs. Additional studies looking at the effect of the different ICIs are needed to assess the optimal treatment regimen.

The effectiveness of systemic treatment options is summarized in Table 5.

Recommendation:

Triplet therapy (chemotherapy + Atezolizumab + bevacizumab) must be upheld as the new standard of care for persistent, recurrent, or metastatic cervical cancer, regardless of PDL1 expression status, for those eligible for bevacizumab treatment [strength of evidence IIA] (grade of recommendation 1) *.

If triplet therapy is unavailable, pembrolizumab* combined with chemotherapy with or without bevacizumab is recommended for tumors expressing PD-L1 (CPS ≥ 1) [strength of evidence IIA] (grade of recommendation 1).

If triplet therapy is unavailable, for patients without tumor PD-L1 expression but who are eligible for bevacizumab, doublet therapy (combining chemotherapy with bevacizumab) remains the preferred option [strength of Evidence: IIA] (Grade of Recommendation: 1).

The lack of PD-L1 expression in concert with contraindication to bevacizumab stratify patient to chemotherapy only [strength of evidence IIA] (grade of recommendation 1).

*If available.

**Figure 5:**
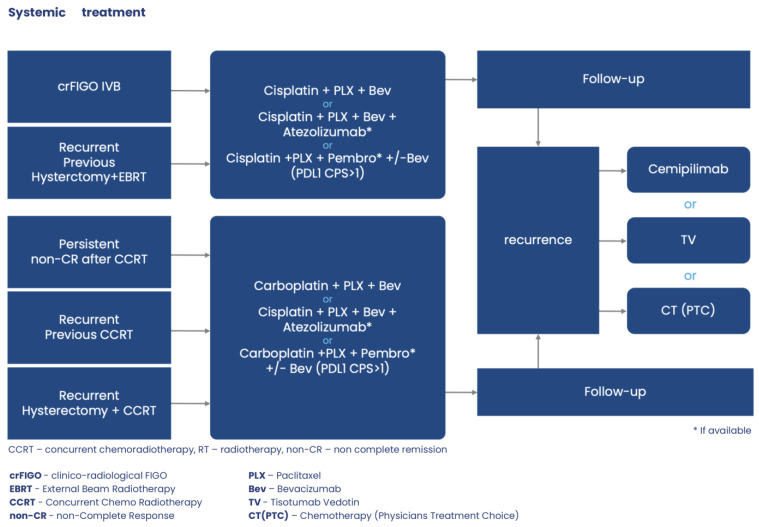
Illustration of the decision tree for systemic treatment of metastatic or persistent/recurrent cervical cancer (mprCC). Detailed data on systemic treatment are described in Supplementary File S6. * If available.

The decision tree for systemic treatment of metastatic, persistent/recurrent cancer (mprCC) illustrates Figure 5.

#### 4.4.2. Second Line Treatment for Recurrent Disease

The EMPOWER trial (NCT 03257267) assessed the efficacy of single-agent cemiplimab, a PD-1-blocking antibody, in improving OS for patients with recurrent or metastatic cervical cancer (rmCC) after first-line platinum-containing chemotherapy [105] [strength of evidence IIA].

Six hundred and eight patients were randomized to receive either cemiplimab (350 mg every 3 weeks) or the investigator’s choice of single-agent chemotherapy. The results showed a longer median OS in the cemiplimab group compared to chemotherapy (12 vs. 8.5 months) (HR = 0.69; 95% CI = 0.56–0.84), regardless of histological subtype. This study represents the largest phase III randomized trial to date demonstrating a significant survival benefit in rmCC post-first-line platinum-containing chemotherapy; thus, cemipilimab is recommended as second-line treatment (if available) (grade of recommendation 1)

The phase III innovaTV 301 trial (NCT04697628) investigated the efficacy of the antibody–drug conjugate (ADC), tisotumab vedotin (TV), in recurrent or metastatic cervical cancer patients who experienced disease progression after chemotherapy [TV] [strength of evidence IIA]. Four hundred and eighty two patients were randomized to receive either TV monotherapy or the investigator’s choice of topotecan, vinorelbine, gemcitabine, irinotecan, or pemetrexed. Notably, 64% and 27.5% of patients had prior bevacizumab or immune checkpoint inhibitor (ICI) therapy, respectively. The results showed a 30% reduction in the risk of death in the TV arm compared to chemotherapy (HR = 0.70; 95% CI = 0.54–0.89), alongside statistically significant improvements in median PFS and OS [106] [strength of evidence IIA]. Hence, TV emerged as a recommended option for rmCC patients who have exhausted first-line treatment options (grade of recommendation 1).

### 4.5. Particular Clinical Situations

#### 4.5.1. Oligometastases

It delineates a distinct form of disseminated cervical cancer (FIGO IVB) in which the primary site coexists with synchronous metastases, numbering up to six, involving hematogenous and/or lymphatic pathways [107,108] [strength of evidence IIIE, IIIE].

Numerous studies indicate that conventional multiagent systemic treatments may not adequately address these cases, with potentially improved outcomes achievable through definitive local therapy [107,108,109] [strength of evidence IIIE, IIIE, IIID].

A significant retrospective study examining over 2800 patients with metastatic cervical cancer at initial diagnosis demonstrated that definitive local therapy, such as radiation therapy or surgery to the primary site (n = 1194), was associated with improved OS (hazard ratio: 0.57; 95% confidence interval, 0.52–0.62; *p* ≤ 0.001), with a median OS of 19.2 months compared to 10.1 months in the conservative therapy cohort (n = 1644). Furthermore, within the definitive local therapy group, the addition of a BRT boost to external beam radiation therapy (EBRT) alone was linked to further improvements in OS (HR: 0.63; 95% CI, 0.54–0.74; *p* < 0.001). Sensitivity analyses supported these findings, indicating that even substantial unmeasured confounding would not negate the significance of the results. Overall, the study concluded that definitive local therapy offers enhanced OS in patients with metastatic cervical cancer, suggesting a promising approach in this setting [109] [strength of evidence IIID].

Another small-scale retrospective cohort study confirmed that definitive chemoradiation (MMRT/VMAT) targeting both the primary site and oligometastases emerges as a feasible option warranting consideration for FIGO IVB patients. This approach analyzed in 60 oligometastatic patients demonstrated promising results with a median PFS of 52.3 months, comparable to systemic treatments such as triplet (CT + Atezo + Bev vs. CT + Bev). Notably, the median OS was not reached. The study also indicated variations in outcomes based on metastatic patterns, with lymphatic metastases associated with better OS compared to hematogenous metastases (3-year OS rates: 57.2% vs. 20%, *p* = 0.017). Additionally, patients with a single metastasis site exhibited more favorable prognoses than those with two or more sites (3-year OS rates: 60.4% vs. 20.6%, *p* = 0.003) [107] [strength of evidence IIIE].

Recommendation:

For patients with oligometastatic cancer, definitive radiation therapy (CCRT + BRT + stereotactic body radiotherapy (SBRT)) targeting both the metastatic and primary sites can be considered, particularly in carefully selected cases. There seems to be flexibility regarding the number of lymphatic metastases sites, potentially making definitive radiation therapy a viable primary treatment option. However, when addressing hematogenous metastases, it is recommended to prioritize cases involving the primary site and a single metastatic site for CCRT + BRT + SBRT [expert opinion] [grade of recommendation 2B].

**Figure 6:**
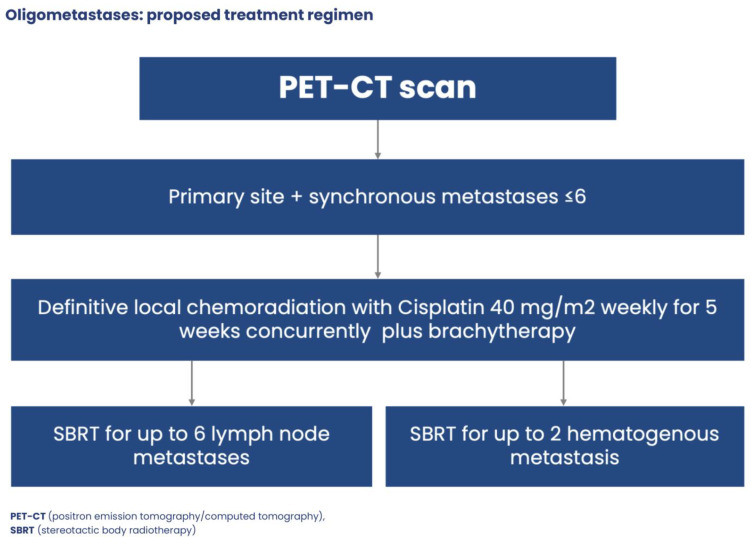
Illustration of the proposed treatment approach for oligometastatic disease.

The proposal treatment approach for oligometastatic desease in cervical cancer illustrates Figure 6.

#### 4.5.2. Fertility Sparing Management

Both conization (CON) and radical trachelectomy (RTr), with or without lymphadenectomy, show promising results as fertility-sparing treatments for early-stage cervical cancer (eCC), particularly in stage IAIB1. This is evidenced by the low relapse rates associated with CON and RTr (recurrence rate of 2.3%, a death rate of 0.7%), along with a notable proportion of women successfully achieving pregnancy (pregnancy rate of 20.5%, a spontaneous abortion rate of 24.0%, and a preterm delivery rate of 26.6%).

From a subgroup analysis, the recurrence rates for stage IA tumors treated with CON and RTr were 0.4% (0.0–1.9%) and 0.7% (0.0–2.3%), respectively; and for stage IB1, they were 0.6% (0.0–2.7%) and 2.3% (0.9–4.1%) [110] [strength of evidence IIID].

Another review of the available trials confirmed that CON or simple trachelectomy (STr) could be performed for IA1 cervical cancer patients with LVSI who want to preserve fertility, although these results are only based on a small number of nonrandomized studies [111] [strength of evidence IVC].

The results of many retrospective studies have revealed that in small-volume, low-risk, early-stage cervical cancer (defined as measuring < 2 cm with <50% stromal invasion), the probability of parametrial extension is very low and even less than 1% concluded from a 1000 patient retrospective review analysis, which testified indirectly the security of CON [112] [strength of evidence IVB].

For women with lesions > 2 cm who desire to preserve fertility, the available treatment options are limited in terms of safety. The primary approaches involve either upfront RTr or neoadjuvant chemotherapy (NACT) followed by fertility-preserving surgery.

The studies investigating these methods are constrained by their retrospective nature, revealing a 6% risk of recurrence and a 2% mortality rate [113] [strength of evidence IVD].

The ongoing CONTESSA/NEOCON-F trial aims to provide comprehensive data on the efficacy of NACT followed by fertility-preserving surgery in young women diagnosed with cervical cancer (lesions ranging from 2 to 4 cm) [114].

Recommendation:

For patients in stage IA or IB1 with <50% stromal invasion (<10 mm), CON or STr appears more suitable due to its lower rates of miscarriage and preterm delivery, attributed to the minimal damage inflicted on the cervical and parametrial tissues, while still ensuring favorable oncological outcomes [strength of evidence IIID, IVC] (grade of recommendation 2B).

For stage IB1 with >50% stromal invasion (>10 mm) RTr with SLNB or PLND is the preferred conservative management [strength of evidence IIID] (grade of recommendation 2A).

For stage > IB2, it is imperative to approach fertility-sparing management (RTr or NACT followed by fertility-preserving surgery) with extreme caution until the results of CONTESSA trial are published [expert opinion–strength of evidence V] (grade of recommendation 2B).

**Figure 7:**
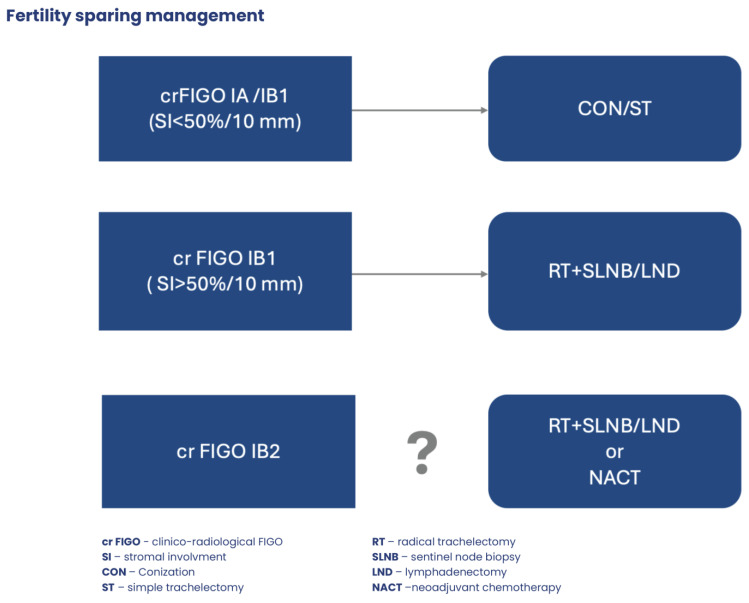
Illustration of the recommended management for fertility-sparing treatment.

The recommended algorithm for fertility sparing treatment illustrates Figure 7.

## 5. Follow-Up

### 5.1. Assessment of Initial Treatment

A complete response (CR) is established when there is no evidence of disease detected three months after the completion of treatment (surgery/surgery plus radiotherapy/radiotherapy), assessed through clinical and radiologic evaluations. If there is evidence of persistence or progression at this point, the treatment is considered “non-CR”, indicating persistent disease. Recurrent disease, on the other hand, signifies the appearance of a new tumor following complete remission [115] [strength of evidence IIIE].

Recommendation: Clinical and radiologic evaluations are mandatory three months after the completion of initial treatment to detect cases of persistent disease*. Any identified instances should be promptly referred for immediate appropriate systemic treatment (grade of recommendation 2A).

*In some cases.

### 5.2. Follow-Up for CR Patients—Evidence

A comprehensive systematic review encompassing seventeen studies, which examined follow-up strategies for women who remained disease-free following primary treatment for cervical cancer, yielded the following key findings:

1. Across nine studies reporting data, a significant majority (62–89%) of cervical cancer recurrences were identified within the initial 2 years post-primary treatment. Furthermore, in six studies, at least 89% of recurrences were detected within a 5-year timeframe.
2. Of the seventeen retrospective studies, fifteen provided insights into whether recurrences were symptomatic or asymptomatic. Approximately two-thirds of patients presented with symptoms, ranging from 46% to 87%, while roughly one-third were asymptomatic, with proportions ranging from 4% to 54%.
3. Scheduled follow-up visits exhibited variability, ranging from a minimum of 9 to a potential maximum of 28 visits over a 5-year period. Most studies outlined similar intervals: follow-up appointments every 3–4 months during the initial 2 years, transitioning to semiannual visits for the subsequent 3 years, and then annual assessments extending to year 10 or discharge.
4. Although not uniformly reported, physical examination and vaginal vault cytology emerged as the most commonly utilized follow-up tests across the seventeen studies. On average, physical examination detected recurrences in 52% of cases, while vaginal vault cytology identified recurrences in approximately 6%.
5. Among the studies reporting on the routine use of additional diagnostic modalities such as chest radiography, abdominal and pelvic ultrasonography, PET, CT, magnetic resonance imaging, intravenous pyelography, or tumor markers, there was a lack of consistency in reporting. Moreover, the impact of asymptomatic recurrence detection on survival remained unclear [116] [strength of evidence IIIA].

The results of this systematic review have shown that the optimal frequency of follow-up visits and the specific parameters to assess during these visits have yet to be definitively established. A prospectively designed study is essential not only to establish a comprehensive checklist for follow-up visits but also to validate the significance of early detection on survival rates. Such research is crucial for guiding clinicians in developing evidence-based follow-up protocols that optimize patient outcomes.

### 5.3. Follow-Up Recommendations

#### 5.3.1. Schedule 

Based on the available data, it is advisable to conduct regular reviews every 3 to 6 months within the initial 2-year period post-treatment, followed by semi-annual check-ups for the subsequent 3 years. This recommendation is grounded in evidence indicating that the majority of recurrences manifest within 36 months post-first-line treatment completion [116] [strength of evidence IIIA] (grade of recommendation 2A).

However, recurrence is often not identified during routine follow-up but rather more frequently following unscheduled clinic visits [117] [strength of evidence IVC].

#### 5.3.2. Symptoms

Patients commonly exhibit symptoms upon recurrence, such as vaginal bleeding, low-back pain radiating to a leg, and unexplained weight loss [116] [strength of evidence IIIA].

Thus, it is imperative to thoroughly counsel patients regarding these symptoms (grade of recommendation 2A).

#### 5.3.3. Follow-Up Tests

The role of vaginal vault or cervical cytology in follow-up remains a subject of debate, as retrospective studies have highlighted limited utility. Some experts even question its use, given the low cytology detection rates for recurrence observed in these studies (ranging from 0% to 17%). Moreover, a cytological atypical feature rarely serves as the sole indicator of disease recurrence [116,118] [strength of evidence IIIA, V].

The serum SCC-Ag consistently correlates with both recurrence and mortality in newly diagnosed cervical cancer cases. Therefore, it proves to be a valuable marker for monitoring disease progression in patients with cervical cancer [119] [strength of evidence IIIA].

In a study with a sample size of 75, elevated serum levels of SCC-Ag and high-sensitivity C-reactive protein (hsCRP) were found to be strongly associated with increased odds of disease recurrence (*p* = 0.003 and *p* < 0.001, respectively). The combined diagnostic accuracy of these biomarkers was 0.87 (95% CI: 0.805 to 0.935). However, among the seven other biomarkers tested in the same study (CA-15.3, CA-125, CEA, CYFRA 21-1, IL-6, TNF-α, and VEGF), none significantly contributed to predicting recurrence [120] [strength of evidence IIIA].

Physical examination, with or without cytology, along with serum SCC-Ag, with or without hsCRP testing, are recommended for follow-up consultations (grade of recommendation 2A).

In cases where recurrent disease is suspected, it is recommended to perform a biopsy to confirm recurrence, alongside imaging modalities such as MRI, PET, or CT scans. These diagnostic tools aid in evaluating the extent of the disease and guiding subsequent treatment decisions (grade of recommendation 2B).

## Figures and Tables

**Table 1:** Grading criteria according to the Agency for Health Technology Assessment and Tariff System (AOTMiT) guidelines.

Study Type	Grade	Subtype Description
RTC systematic review	IA	Meta-analysis based on RTC systematic review results
	IB	RCT systematic review without meta-analysis
Experimental study	IIA	Well-conducted randomized-controlled trial, including pragmatic randomized-controlled trial
	IIB	Well-conducted clinical-controlled trial with pseudorandomization
	IIC	Well-conducted clinical-controlled trial withoutrandomization
	IID	One-arm study
Observational study with control group	IIIA	Systematic review of observational studies
	IIIB	Well-conducted prospective-cohort studies with simultaneous control group
	IIIC	Well-conducted prospective-cohort studies with historic control group
	IIID	Well-conducted retrospective-cohort studies with simultaneous control group
	IIIE	Well-conducted case–control study (retrospective)
Descriptive study	IVA	Case series—pretest/post-test *
	IVB	Case series—post-test **
	IVC	Other study of a group of patients
	IVD	Case report
Expert opinion	V	Expert opinions based on clinical experience and reports from expert panels

* Pretest/post-test—a study where measurements are taken both before and after the assessed intervention. ** Post-test—a study where measurements are taken only after the intervention.

**Table 2:** Polish Society of Gynecological Oncology (PSGO) recommendation classification system.

Grade of Recommendation	Grading Criteria (Strength of Evidence)
Grade 1	Strength of evidence I or II (unanimity of experts) *
Grade 2A	Strength of evidence III (unanimity of experts) *
Grade 2B	Strength of evidence IV or V (unanimity of experts) * or strength of evidence III (no unanimity of experts) *
Grade 3	Every strength of evidence, when PSGO development group believes that the procedure can be used under certain conditions, but is not appropriate (unanimity) *

* Unanimity = >85% of development group members agree.

**Table 3:** Histopathological classification of cervical cancers.

Cancer Type	ICD-O	Frequency	Definition	Prognosis
Squamous cell tumors (SCCs)				
Squamous cell carcinoma, HPV-associated	8053/3	90–95% of cervical SCCs	HPV-associated squamous tumor with stromal or exophytic-type invasion. It is caused by high-risk HPV infections, with HPV 16 and 18 contributing to 70% of all SCCs. Very rarely, low-risk HPV genotypes, such as 6 and 11, may be the sole cause of cervical SCC. It develops from high-grade squamous intraepithelial lesions due to high viral oncogenes E6 and E7 expression. Over 70% of HPV-associated SCCs present genomic alterations in PI3K/MAPK or TGF-β signaling pathways. Mutations occur also in HER3, CASP8, and TGFBR2 genes. Almost all HPV-associated SCC cells show solid and diffuse p16 overexpression in nuclei and the cytoplasm.	Favorable prognosis. Histological patterns, HPV type, and grading do not seem to have prognostic implications.
Squamous cell carcinoma, HPV-independent	8086/3	5–7% of cervical SCCs	HPV-independent SCCs harbor a higher rate of abnormal p53 staining suggestive of mutation and are frequently of the keratinizing type. This type of SCC is often associated with KRAS, ARID1A, and PTEN mutations. Its macroscopic appearance does not differ from that of HPV-associated cancers. HPV-independent cancers are morphologically undistinguishable from their HPV-associated counterparts, and the lack of HPV infection is necessary for diagnosis. The lack of p16 immunostaining is an acceptable surrogate biomarker.	Unfavorable prognosis due to late diagnosis and frequent lymph node metastasis.
Squamous cell carcinoma, NOS	8070/3	-	As there is no difference in the treatment of HPV-associated and HPV-independent cervical SCC, a morphological diagnosis of squamous cell carcinoma NOS is acceptable if p16 immunostaining or HPV testing is not available.	-
Glandular tumors				
Adenocarcinoma in situ (AIS), HPV-associated	8140/2 8483/2	-	Neoplasm associated with high-risk HPV infections, predominantly HPV16, HPV18, or HPV45. AIS replaces epithelium and is confined to the pre-existing glandular architecture. Histological findings include columnar cells, pseudostratified and hyperchromatic nuclei, apical mitotic figures (floating mitoses), and basal karyorrhexis. AIS shows strong and diffuse p16 staining and increased Ki-67 proliferation index. Typically, AIS lacks PR and ER expression.	Favorable prognosis (T0N0M0).
Adenocarcinoma, HPV-associated	8140/3 8483/3	5% of cervical cancers	Glandular tumor with stromal invasion or exophytic expansile-type invasion, associated with high-risk HPV. The relative frequency of cervical adenocarcinomas increased to 10–25% as a result of the treatment of squamous precancers. Coinfection of multiple HPV genotypes occurs in 10% of adenocarcinomas. Gross lesions present as an exophytic mass or ulceration in the distal cervix. HPV-associated endocervical adenocarcinoma often presents apical mitoses and karyorrhexis, as well as enlarged, elongated, and hyperchromatic nuclei. In total, 95% of HPV-associated carcinomas show diffuse p16 staining. P16 negativity may derive from methylation-induced inactivation. KRAS and PIK3CA mutations are frequent and associated with destructive growth.	The prognosis depends on the stage; the median 5-year overall survival is 77%.
Adenocarcinoma in situ, HPV-independent (also gastric-type adenocarcinoma in situ)	8140/2 8484/2	-	A non-invasive glandular neoplasm unrelated to HPV, characterized by gastric type. The lesion of unknown etiology is localized typically proximally to the transformation zone. The tumor is characterized by cuboidal to columnar cells with distinct cell borders, vacuolated cytoplasm, and nuclear atypia. PAX8 and CDX2 are often positive; ER and PR are usually harmful—no or patchy p16 expression. Abnormal p53 staining suggests the diagnosis.	Unknown behavior. Complete excision is advised.
Adenocarcinoma, HPV-independent, gastric type	8482/3	10–15% of cervical adenocarcinomas	Invasive adenocarcinoma showing gastric differentiation unrelated to HPV infection. They can occur in Peutz–Jeghers syndrome (germline STK11 mutation); TP53 mutation is frequent. It lacks estrogen/progesterone receptors. It seems to derive from lobular endocervical glandular hyperplasia. Tumors are usually large, can be polypoid or ulcerated, and impart a barrel shape to the cervix. They are characterized by glandular cells with abundant clear or pale eosinophilic cytoplasm and distinct cell borders. They show abnormal p53 staining and a lack of p16 overexpression.	Aggressive behavior and poor prognosis.
Adenocarcinoma, HPV-independent, clear cell type	8310/3	3–4% of cervical adenocarcinomas	Malignant glandular neoplasm comprises uniform, precise, eosinophilic, flat, or cuboidal cells arranged in one or more patterns: tubulocystic, papillary, or solid. Sporadic tumors occur in the endocervix, while tumors associated with in utero diethylstilbestrol (DES) exposure occur in the ectocervix (rare *POLE* mutation). They are not related to high-risk HPV inventions. Tubulocystic, papillary, or solid growth patterns. Negative for ER or HPV.	Favorable prognosis and low recurrence rates.
Adenocarcinoma, HPV-independent, mesonephric type	9110/3	<1% of cervical adenocarcinomas	Rare, nonhuman papillomavirus-associated cervical neoplasm likely deriving from mesonephric (Wolffian) remnants. The tumor is usually deeply located and shows varied growth patterns, including solid, cystic, spindle cell, and mesonephric hyperplasia-like growth. Most harbor frequent KRAS mutations and 1q chromosome gain, as well as sporadic TP53 or CTNNB1 mutations. Most tumors have a low mutation burden and lack microsatellite instability.	Aggressive behavior and frequent recurrence.
Other adenocarcinomas of the uterine cervix	8140/3	<1% cervical adenocarcinomas	This term includes endometrioid adenocarcinoma of endocervix, endometrioid adenocarcinoma and serous carcinoma secondarily involving the cervix, and adenocarcinoma NOS, which were included in the previous WHO classification. Endometrioid adenocarcinoma of the endocervix is thought to arise in endometriosis. Adenocarcinoma NOS is a heterogenous category. Those types of cancer are typically HPV-independent. A p16, ER, and GATA3 panel may be used to narrow differential diagnosis. If performing the additional tests is impossible, the terms “HPV-associated (or HPV-independent) adenocarcinoma NOS” are acceptable.	Uncertain prognosis due to lack of uniform criteria.
Adenocarcinoma in situ, NOS	8140/2	1% of cervical non-invasive lesions	Presents variable histologic features based on histological type. Usually associated with HPV infections. Negative p16 staining indicates the lack of association with HPV. Mostly in young patients. In 50%, it coexists with high-grade squamous intraepithelial lesions.	Excellent prognosis in most cases.
Other epithelial tumors				
Carcinosarcoma	8980/3		Carcinosarcoma is a biphasic malignant neoplasm composed of epithelial and mesenchymal cells. It occurs after menopause and presents as a large polypoid mass, often with necrosis and hemorrhage. Carcinosarcomas are usually associated with high-risk HPV infection, predominantly 16 and 18 subtypes. Carcinosarcomas are stages of cervical cancer.	Carcinosarcomas present at an earlier stage than cervical cancers and may have a better prognosis.
Adenosquamous and mucoepidermoid carcinomas	8560/3 8430/3	5–6% of cervical cancers	Malignant epithelial tumors exhibiting squamous and glandular differentiation. The squamous cells may exhibit abundant clear, glycogen-rich cytoplasm. Both tumor components are usually admixed and should be recognizable without additional stains. Their pathogenesis is associated with HPV 16 and 18 infections. Both cancers show lower ARID1A, f EGFR, and PDGFRA levels than squamous carcinomas. Both tumor components exhibit diffuse p16 staining. The epithelial component is typically positive for CK7, CEA, and PAX8, while the squamous component is p63- and p40-positive.	Traditionally, aggressive behavior. Newer studies indicate a prognosis similar to SCCs.
Adenoid basal carcinoma	8098/3	<1% of all cervical cancers	An epithelial tumor comprises morphologically bland, small, and rounded nests of basaloid cells and is usually associated with high-risk HPV infections. Most adenoid basal carcinomas are associated with a high-grade squamous intraepithelial lesion or an invasive carcinoma of another type. Diagnosis of a pure adenoid basal carcinoma must include examining the whole tumor. If another invasive carcinoma is present, a mixed tumor should be reported. Tumor cells are positive for cytokeratins, p16, and p63.	No known metastatic potential. The prognosis of mixed tumors depends on the features of the other components.
Carcinoma of the uterine cervix, unclassifiable	8020/3	<1% of all cervical cancers	Malignant epithelial tumor of the cervix that cannot be further subclassified. Diagnosis requires the exclusion of other primary and metastatic tumors. <Mt cases are HPV-associated or block-type p16-positive.	Similar to most cervical SCCs.
Mixed epithelial and mesenchymal tumors				
Adenosarcoma	8933/3	0.16% of all cervical cancers; 10% adenosarcomas of female genital tract	A rare, mixed lesion with malignant mesenchymal and benign glandular components that occurs mainly in younger patients. It has an unknown etiology and usually low malignant potential. Characterized by leaf-like glands composed of bland epithelium and condensed periglandular stroma with atypia and mitotic activity. Stromal cells may lack CD10 and PR expression when sarcomatous overgrowth is present. Recurrence may consist solely of sarcomatous components.	Favorable prognosis.
Germ cell tumors				
Germ cell tumors of the uterine cervix	9064/3	Rare	It is characterized by a polypoid, friable tumor deriving from primordial germ cells. It may present in various histological subtypes, including mature teratoma NOS, dermoid cyst NOS, endodermal sinus tumor, yolk sac tumor NOS, and choriocarcinoma NOS. Metastasis from the ovary must be excluded.	Mature teratomas and yolk sac tumors have a good prognosis. Choriocarcinomas may follow an aggressive course.

**Table 4:** Staging of cervical tumors according to the International Federation of Gynecology and Obstetrics (FIGO 2018) and the American Joint Committee on Cancer (AJCC).

Clinical and/or Radiological Features	TNM	FIGO
Cervical carcinoma confined to the cervix (without extension to uterine corpus)	T1/N0/M0	I
Invasive carcinoma diagnosed only by microscopy, stromal invasion with a maximum depth of 5.0 mm measured from the base of the epithelium, and horizontal spread of 7.0 mm or less; vascular space involvement, venous or lymphatic, does not affect classification	T1A	IA
Measured stromal invasion no greater than 3.0 mm and lateral spread no greater than 7.0 mm	T1A1	IA1
Measured stromal invasion greater than 3.0 mm and no greater than 5.0 mm, and horizontal spread no greater than 7.0 mm	T1A2	IA2
Clinically visible lesion confined to the cervix or microscopic lesion greater than T1a or IA2	T1B	IB
Clinically visible lesion no greater than 2.0 cm in greatest dimension	T1B1	IB1
Clinically visible lesion no greater than 4.0 cm in greatest dimension	T1B2	IB2
Clinically visible lesion greater than 4.0 cm in greatest dimension	T1B3	IB3
Cervical carcinoma invades beyond the uterus but not the pelvic wall or lower third of vagina	T2/N0/M0	II
Tumor without parametrial invasion	T2A	IIA
Clinically visible lesion no greater than 4.0 cm in greatest dimension	T2A1	IIA1
Clinically visible lesion greater than 4.0 cm in greatest dimension	T2A2	IIA2
Tumor with parametrial invasion	T2B	IIB
Tumor extends to pelvic wall, involves lower third of vagina, causes hydronephrosis, or a combination of all symptoms, or non-functioning kidney or involvement of pelvic and/or para-aortic lymph nodes, irrespective of tumor size and extent (with r and p notations) *	T3, N0 or any T, N (+)	III
Tumor involves lower third of vagina, without extending to the pelvic wall	T3A	IIIA
Tumor extends to pelvic wall, causes hydronephrosis or non-functioning kidney, or both	T3B	IIIB
Pelvic lymph node metastasis only	Any T/N1	IIIC1
Para-aortic lymph node metastasis	Any T/N2	IIIC2
Tumor invades mucosa of bladder or rectum, extends beyond the true pelvis, or both (bullous oedema is not sufficient to classify a tumor as T4 or IV)	T4	IV
Spread to adjacent pelvic organs: tumor invades mucosa of bladder or rectum (bullous oedema is not sufficient to classify a tumor as T4A or IVA)	T4A	IVA
Spread to distant organs	Any T/any N/M1	IVB

* Adding notation of r (imaging) and p (pathology) to indicate the findings that are used to allocate the case to Stage IIIC. Example: If imaging indicates pelvic lymph node metastasis, the stage allocation would be IIIC1r, and if confirmed by pathology, it would be IIIC1p.

**Table 5:** Systemic treatment options for metastatic, persistent, recurrent cervical cancer.

	GOG 240	Keynote-826	BEAtcc
Treatment	CT + Bevacizumab	CT + Pembrolizumab with or without Bevacizumab vs. CT + Bev	CT + Atezo + Bev vs. CT + Bev
Median OS	17.0 mo, HR 0.71	24.4 mo, HR 0.64	32.2 mo, HR 0.62 HR 0.68 [95% CI 0.52–0.88]; *p* = 0.0046
ORR	48.0%	68.1% in PD-L1 + ≥1%	84% independently of PD-L1
Citation	[97,98]	[100,101]	[103,104].

## Data Availability

No new data were created or analyzed in this study. Data sharing is not applicable to this article.

## Supplementary Materials

The following supporting information can be downloaded at: https://www.mdpi.com/article/10.3390/jcm13154351/s1, References [121,122,123,124,125,126,127,128,129,130] are cited in Supplementary Materials. File S1: Core needle biopsy specimen and postoperative specimen final report. File S2: Types of hysterectomy. File S3: Technique of sentinel lymph node identification for cervical cancer. File S4: Adjuvant pelvic radiotherapy. File S5: Curative radical radiotherapy. File S6: Systemic treatment.
