# Supplementary material for: The Polish Society of Gynecological Oncology Guidelines for the Diagnosis and Treatment of Cervical Cancer (v2024.0)
Source: J Clin Med. 2024 Jul 25;13(15):4351. doi: 10.3390/jcm13154351 (PMC11313441; doi:10.3390/jcm13154351)
Supplement: Supplementary file 1 [file jcm-13-04351-s001.zip › PSGO, File S1.pdf]

File S1: Core needle biopsy specimen and postoperative specimen final report.

The current guidelines from the Polish Society of Gynaecological Oncology (PSGO 2024v1) recommend core needle biopsy (CNB) for macroscopic lesions up to 4 cm in greatest dimension. The CNB specimen report should at least include a description of the LVSI and if feasible the depth of stromal invasion.

These factors are pivotal in determining the suitability of a patient with a tumor < 2 cm for a simple hysterectomy (Shape trial 2023) or for those with tumors > 2 cm < 4 cm for either surgery or radiotherapy (Sedlis criteria).

Core biopsy is considered adequate if the histological specimen is > 2mm in maximum diameter. Any biopsy that shows an abnormality should be considered adequate [strength of evidence V] (grade of recommendation 2B).

PSGO will collaborate with PTP to integrate the core needle biopsy (CNB) specimen report into the Guidelines for pathology departments/laboratories.

Currently the Polish Society of Pathology (PTP) (Pathology: standards and examples of good practice and differential diagnosis. Guidelines for pathology departments/laboratories. Polish Ministry of Health) recommends that the postoperative final report should include at least:

Conization:

1) the type of collected material and performed procedure (ICD-9);

2) description of macroscopic examination, including:

a) identified pathological changes and their localization;

b) information about the appropriate marking of the sample for its correct orientation, e.g. with a thread (at least one conventional landmark, usually 12:00);

c) lesion greatest dimension and additional dimensions[1];

3) description of microscopic examination, including:

a) histological diagnosis according to the WHO classification[2] and ICD-O code;

b) primary tumor stage (pT, FIGO; if possible);

c) grade (G1-G3);

d) depth of stromal invasion;

e) the status of endocervical, exocervical and deep margins;

f) assessment of lymph-vascular space invasion (LVSI);

g) collateral changes/lesions;

Radical hysterectomy:

1) the type of collected material and performed procedure (ICD-9);
2) description of macroscopic examination, including:
a) tumor size (biggest diameter and additional diameters);
b) information regarding appropriate marking of the sample for its correct orientation;
c) tumor localization;
3) description of microscopic examination, including:
a) histological diagnosis according to the WHO classification and ICD-O code.
b) grade (G1-G3)[3];
c) primary tumor stage (pT, FIGO);
d) margins[4]: distance from invasive carcinoma, distance from preinvasive carcinoma, margins with invasion;
e) minimum distance of uninvolved cervical stroma;
f) assessment of lymph-vascular space invasion (LVSI);
g) depth of stromal invasion
h) lymph node status[5]; isolated tumor cells and the method of detection (pN, FIGO);
i) description of coexisting pathology.
4) HPV status and the results of immunostaining[6].

Furthermore, to qualify for treatment with pembrolizumab, PSGO recommends testing for PD-L1 expression in all cases of persistent, recurrent, or metastatic cervical cancer in patients that did not receive previous chemotherapy.

How to calculate CPS score for PD-L1 [121]:

A minimum of 100 viable tumor cells must be present in the PD-L1–stained slide (sectioned tumor biopsy or resection tissue) for the specimen to be considered adequate for evaluation. Tumor cells must show partial or complete membrane staining (≥1+) to be counted as "stained," whereas immune cells are counted if there is any staining. The combined positive score (CPS) is defined as the proportion of all PD-L1–positive cells to the total number of PD-L1–positive and PD-L1–negative tumor cells in a sample.

Starting from April 2024, patients with advanced cervical cancer and CPS >1 will qualify for treatment with pembrolizumab as first-line therapy. Therefore, reporting CPS scored in ranges <1, 1–<10, and ≥10 is strongly recommended.

After a hysterectomy, determining the need of adjuvant treatment for patients relies on the Sedlis criteria. These criteria are established through a scoring system, as outlined below:

- Presence of lymphovascular space invasion (LVSI) alongside deep (outer third) cervical stromal invasion, regardless of tumor size.
- Presence of LVSI alongside middle (one-third) stromal invasion and a tumor size of 2 cm or greater.
- Presence of LVSI alongside superficial (inner third) stromal invasion and a tumor size of 5 cm or greater.
- Absence of LVSI but with deep or middle cervical stromal invasion and a tumor size of 4 cm or greater.

It is crucial to include these features in the pathological report to guide treatment decisions effectively. Thus PSGO will collaborate with PTP to integrate the categorization of stromal invasion proposed by Sedlis into pathological report.

[1] All dimensions should be precisely described due to their strong influence on clinical staging.

[2] Reliable assessment of histological subtype of cancer may require p16 and Ki-67 staining.

[3] Due to its lack of prognostic significance in squamous cell carcinoma of the uterine cervix, grade is not obligatorily described. In adenocarcinoma, grade (based on the morphology and atypical features of cell nucleus) is associated with prognosis. G1 - scarce solid areas and small/moderate atypia; G2 - morphology between G1 and G3; G3 - solid areas and significant nuclear atypia.

[4] Margins should be described as free, involved, or unable to be assessed. It must be precisely stated which margins is involved and whether the invasion is focal (aggregated) or diffuse. If the tumor is near the margin, but the margins remains free, the distance between the invasion and the assessed margin should be provided.

[5] Regional lymph nodes include parametrial, paracervical, obturator, presacra, sacral, external iliac, common iliac and internal iliac lymph nodes. The report should include sentinel lymph node status, the total number of nodes found, the number of positive lymph nodes, and the presence of extranodal extension (list for all separates sites). Micrometastases (>0.2 mm and up to 2 mm) are reported as pN1(mi). Isolated tumor cells ≤ 0.2 mm in regional nodes should be reported as pN0 (i+).

[6] P16 is a useful marker of high-risk HPV infections. Specific situation may require additional tests. Markers, such as estrogen receptor, p16, vimentin and CEA can be used to differentiate cervix adenocarcinoma and endometrial adenocarcinoma, especially with mucous differentiation.
