# Supplementary material for: The Polish Society of Gynecological Oncology Guidelines for the Diagnosis and Treatment of Cervical Cancer (v2024.0)
Source: J Clin Med. 2024 Jul 25;13(15):4351. doi: 10.3390/jcm13154351 (PMC11313441; doi:10.3390/jcm13154351)
Supplement: Supplementary file 1 [file jcm-13-04351-s001.zip › PSGO, File S2.pdf]

File S2: Types of hysterectomy.

Piver-Rutledge-Smith classification [122]

Class 1 Extrafascial hysterectomy: the fascia of the cervix and lower uterine segment is removed with the uterus; the uterine artery is ligated close to uterus; uterosacral and cardinal ligaments are left intact; and the vagina is not resected.

Class 2 Modified radical hysterectomy (Wertheim): the ureters are dissected in the paracervical region but not resected from the vesicouterine ligaments; uterine arteries are ligated when they traverse the ureters; uterosacral ligaments are excised midway from their sacral insertion; cardinal ligaments are resected up to their medial half; and the upper third of the vagina is resected.

Class 3 Radical hysterectomy: uterine arteries are ligated at their origin from the superior vesical or internal iliac arteries; uterosacral and cardinal ligaments are resected at their attachments to the sacrum and pelvic sidewall; and the upper half of the vagina is resected

Class 4 Radical hysterectomy: ureters are completely dissected from the vesicouterine ligaments; superior vesical arteries are sacrificed; and the proximal three quarters of the vagina are resected

Class 5 Radical hysterectomy: bladder is partially resected, or distal ureter is resected with ureteral reimplantation into the bladder.

Querleu Morrow classification [123]

Type A Extrafascial hysterectomy: the position of the ureters is determined by palpation or direct vision (after opening of the ureteral tunnels) without freeing the ureters from their beds; the paracervix is transected medial to the ureter, but lateral to the cervix; the uterosacral and vesicouterine ligaments are not transected at a distance from the uterus; and vaginal resection is limited (<10 mm).

Type B Radical hysterectomy: uterosacral and vesicouterine ligaments are partially resected; the ureter is unroofed and rolled laterally, permitting transection of the paracervix at the level of the ureteral tunnel; the caudal (posterior, deep*)* neural component of the paracervix caudal to the deep uterine vein is not resected; and at least 10 mm of the vagina from the cervix or tumour is resected.

Type C Radical hysterectomy: the uterosacral ligament is transected at the rectum and vesicouterine ligament at the bladder; the ureter is mobilized completely; and 15 to 20 mm of the vagina from the tumour or cervix and the corresponding paracolpos is resected.

Type D Additional ultraradical procedures, mostly indicated at the time of pelvic exenteration; type D1 is resection of the entire paracervix at the pelvic sidewall along with the hypogastric vessels, exposing the roots of the sciatic nerve; and type D2 is the same as type D1 plus resection of the entire paracervix with the hypogastric vessels and adjacent fascial or muscular structures.
