# Supplementary material for: The Polish Society of Gynecological Oncology Guidelines for the Diagnosis and Treatment of Cervical Cancer (v2024.0)
Source: J Clin Med. 2024 Jul 25;13(15):4351. doi: 10.3390/jcm13154351 (PMC11313441; doi:10.3390/jcm13154351)
Supplement: Supplementary file 1 [file jcm-13-04351-s001.zip › PSGO, File S3.pdf]

File S3: Technique of sentinel lymph node identification for cervical cancer [124]

Lymphoscintigraphy

This procedure involves injecting 0.1–0.5 mCi radiolabeled filtered Tc 99m microsulfur colloid in a volume of 0.1–0.5 ml directly into the cervix following a speculum examination. The injection is administered using a spinal needle in the four quadrants nearest to the area of normal cervix/tumor interface, or alternatively, injections can be made at the 3 and 9 o'clock positions. For patients who have previously undergone a cone biopsy, the injection is directed into the bed of the cone, specifically targeting the cervical stroma. The administration of the radiolabeled injection and the disposal of syringes and other materials should be managed by the Nuclear Medicine Department.

The injection of methylene blue is performed in the operating room while the patient is under anesthesia. It is injected into the cervix adjacent to the lesion. A tenaculum may be utilized to aid in the stromal injection. The 4 ml of blue dye can be divided into four separate injections, one in each quadrant of the cervix (1 ml each). Alternatively, injections can be administered at the 3 and 9 o'clock positions to avoid staining of the bladder flap by the 12 o'clock injection, which corresponds more closely to the parametria.

Sentinel lymph node mapping algorithm [125].

Excision of all mapped SLN[a]

Submit for ultrastaging if negative H&E

↓

Any suspicious nodes must be removed regardless of mapping.

↓

If there is no mapping on a hemipelvis, a side specific LND[b] is performed.

Parametrectomy is performed en block with resection of the primary tumor.

a Intracervical injection with isosulfan blue dye, 99m Technetium, or both;

b Including interiliac/subaortic nodes;

Blue dye/technetium can be safely replaced with ICG or carbon nanoparticle.

The detection rate of SLN mapping in the unilateral pelvis was median 95.7% and 100% and in the bilateral pelvis was median 80.4% and 90% for technetium-99 m (Tc) with/without blue dye (Tc w/wo BD) and indocyanine green (ICG) alone, respectively. The sensitivity and specificity of each tracer were high; the area under the curve of each tracer was 0.988 (Tc w/wo BD), 0.931 (BD w/wo Tc), 0.966 (ICG), and 0.977 (carbon nanoparticle) [126] [strength of evidence IB].

These data indicate comparable efficacy between indocyanine green (ICG) alone, carbon nanoparticle alone and technetium with/without blue dye. However, it is important to consider that technetium, if its logistical implementation issues are resolved, offers greater flexibility due to the possibility of reinjection.
