# Supplementary material for: The Polish Society of Gynecological Oncology Guidelines for the Diagnosis and Treatment of Cervical Cancer (v2024.0)
Source: J Clin Med. 2024 Jul 25;13(15):4351. doi: 10.3390/jcm13154351 (PMC11313441; doi:10.3390/jcm13154351)
Supplement: Supplementary file 1 [file jcm-13-04351-s001.zip › PSGO, File S4.pdf]

File S4: Adjuvant pelvic radiotherapy

With the advancements in image-based treatment planning utilizing CT and MRI, soft tissue regions at risk, such as parametrial and vaginal tissue, and pelvic lymph nodes can be effectively treated while minimizing exposure to adjacent tissues like the bladder, rectum, and bowel. This should be achieved through techniques such as intensity-modulated radiotherapy (IMRT) including VMAT (*Volumetric Modulated Arc Therapy*) a dynamic arc radiation technique and IGRT (*Image-guided radiation therapy*). IGRT facilitates X-rays and CT (Computed Tomography), CBCT (Cone Beam Computed Tomography), or MR (Magnetic Resonance) imaging on the therapeutic device before each radiation fraction to ensure accurate patient positioning, verify the position of internal organs near the treated area, and localize the tumor/treated organs.

Adjuvant radiotherapy (patients with Sedlis criteria)

- Pelvis EBRT + BRT

Generally, postoperative radiation therapy comprises external beam radiotherapy (EBRT) and vaginal cuff brachytherapy (BRT).

Pelvis irradiation includes vaginal cuff, parametria and obturator, iliac internal, external, common iliac and presacral lymph nodes as clinical target volume (CTV). The planning target volume (PTV) margin to CTV, if no measurement data are available at the center, is 7 mm for nodal areas. If necessary paraaortic lymph nodes are irradiated.

The recommended dose for the pelvis is 45 Gy/25 fractions or 46 Gy/23 fractions, and for the pelvis with para-aortic lymph nodes, it is 45 Gy/25 fractions. Vaginal cuff brachytherapy HDR (high dose rate) to 12-14 Gy in two fractions, treatment plans based on CT are recommended.

*Adjuvant concurrent chemoradiotherapy /CCRT/ (patients with Peters crite*ria)[86].

- Pelvis +/- PAO EBRT + BRT + (cisplatin) DDP

After primary hysterectomy with lymphadenectomy, the presence of one or more pathological risk factors according to the Peters criteria (positive pelvic lymph nodes and/or positive margins and/or microscopic involvement of the parametrium) requires the use of adjuvant chemoradiotherapy and vaginal cuff brachytherapy.

Concurrent chemotherapy to RT

- Cisplatin 40 mg/m^2 i.v. weekly, (or carboplatin at AUC2 weekly in patients with impaired renal function) administered continuously with concurrent radiation therapy; typically 4 to 5 cycles depending on the duration of radiation therapy. [127,128].
- or Cisplatin (70 mg/m^2) i.v. on day 1 plus fluorouracil (1000 mg/m^2) i.v. on days 1-4 as a 96-hour infusion, administered every 3 weeks for four cycles. The first and second cycles are administered concurrently with radiation therapy.[86].

Sequential Chemoradiation (SCRT) according STARS Trial [89].

Sequential radiochemotherapy comprises 4 cycles of chemotherapy administered both before and after radiotherapy (2 cycles preceding and 2 cycles succeeding radiotherapy).

Cisplatin is administered at a dosage of 60 to 75 mg/m^2 on day 1 via a 1-hour IV infusion, while paclitaxel is given at a dosage of 135 to 175 mg/m^2 on day 1 through a 3-hour IV infusion.

Radiotherapy should commence 3 to 6 weeks after the second cycle of chemotherapy, and the third and fourth cycles of sequential chemotherapy ought to commence between 3 and 6 weeks after the completion of radiotherapy.

Radiotherapy: Pelvic radiation should be applied in fractions of 1.8 Gy or 2.0 Gy daily, five times a week, with a total dose ranging from 45 Gy to 46 Gy using external beam radiation.
