# Supplementary material for: The Polish Society of Gynecological Oncology Guidelines for the Diagnosis and Treatment of Cervical Cancer (v2024.0)
Source: J Clin Med. 2024 Jul 25;13(15):4351. doi: 10.3390/jcm13154351 (PMC11313441; doi:10.3390/jcm13154351)
Supplement: Supplementary file 1 [file jcm-13-04351-s001.zip › PSGO, File S5.pdf]

File S5: Curative radical radiotherapy

Radiation therapy consists of external beam radiotherapy /EBRT/ and brachytherapy /BRT/.

- The technique for irradiating the pelvis with or without paraaortic lymph nodes is IMRT (intensity-modulated radiation therapy) - a technique of modulated intensity beams, including VMAT (Volumetric Modulated Arc Therapy) - a dynamic arc radiation technique. The PTV margin to CTV, if there are no measurement data available at the center, is 7 mm for nodal areas and 1-1.5 cm for the uterus.
- Image-guided radiation therapy (IGRT) allows for X-rays and most commonly CT (computed tomography), CBCT (cone beam computed tomography), or MR (magnetic resonance) imaging on the therapeutic device before each radiation fraction to control patient positioning, the position of internal organs near the treated area, and tumor localization.
- The recommended dose for the pelvis is 45 Gy/25 fractions or 46 Gy/23 fractions, and for the pelvis with para-aortic lymph nodes, it is 45 Gy/25 fractions.
- A higher dose is administered simultaneously to enlarged lymph nodes, using the SIB (Simultaneous-integrated boost) technique, typically 60 Gy/25 fractions, or a lower dose is given for massively involved lymph nodes.
- BRT is planned based on pre-treatment MRI, and at least the first fraction of brachytherapy is performed with an in situ applicator, the so-called IGBT (image-guided brachytherapy). Planning complies with GEC-ESTRO recommendations. The remaining fractions may be planned with CT.
- The use of intracavitary-interstitial applications is recommended for patients with advanced disease, large tumors of the cervix poorly responsive to radiochemotherapy, cases with unfavorable anatomy such as asymmetrical invasion or a narrow vagina, contributing to poor dose distribution.

- Recommended dose-volume histograms (dose volume histogram) EQD2 (equivalent doses per fraction of 2 Gy): D90 HR-CTV (high-risk clinical target volume) > 85 Gy, D98 HR-CTV > 75 Gy, D98 GTV (gross tumor volume) > 90-95 Gy, D98 IR-CTV (intermediate-risk clinical target volume) > 60 Gy for α/β=10; and D 2cm3 for the rectum < 70-75 Gy, for the bladder < 80-85 Gy, for the sigmoid and intestines < 70-75 Gy, ICRU (International Commission on Radiation Units and Measurements) rectovaginal point < 65-75 Gy for α/β=3.
- The total treatment time should not exceed 50 days, except in cases complicated by severe acute radiation reaction. • D90 minimal dose delivered to 90% of the target • D 2cm3 minimal doses delivered to the most irradiated 2cm3 parts of the organs • α/β=3 for late normal tissue reactions, α/β=10 for tumor

Concurrent chemotherapy to radiotherapy

- Cisplatin 40 mg/m^2 i.v. weekly, (or carboplatin at AUC2 weekly in patients with impaired renal function) administered continuously with concurrent radiation therapy; typically 5 or 6 cycles depending on the duration of radiation therapy. [128,129,130]

To prevent cisplatin nephrotoxicity, it should be administered following hydration: 10 mmol magnesium sulfate (MgSO4) in 1000 mL sodium chloride 0.9% over 60 minutes. Then administer cisplatin dissolved in 1000 mL sodium chloride 0.9% via IV infusion over 60 minutes. After completing the cisplatin infusion, hydration should be administered: 1000 mL sodium chloride 0.9% over 60 minutes.

Induction chemotherapy + CCRT (INTERLACE) [93]

Induction chemotherapy: carboplatin at AUC2 IV plus paclitaxel at 80mg/m^2 IV every 7 days for weeks 1-6 inclusive, followed by standard chemoradiation in week 7.

The recommended standard chemoradiotherapy regimen in the INTERLACE study comprises:

cisplatin 40 mg/m^2 (capped at a total dose of 70 mg) weekly for a maximum of five weeks. This regimen begins in the first week of radiotherapy or as soon as blood counts have recovered from induction chemotherapy. It is administered alongside external beam radiotherapy (EBRT) followed by brachytherapy.

EBRT is recommended with 40-50.4 Gy in 20-28 fractions, followed by intracavity brachytherapy to achieve a minimum total EQD2 dose of 78-86 Gy to point A.

CCRT + Pembro + Pembro mainteinance KEYNOTE A18 [96]

Pembrolizumab is administered at 200 mg IV every 3 weeks for 5 cycles in combination with standard chemoradiotherapy. Subsequently, there are 15 cycles of pembrolizumab at 400 mg IV every 6 weeks.

The standard chemoradiotherapy regimen consists of 5 cycles of cisplatin (40 mg/m^2) administered IV once weekly, with the possibility of an optional sixth dose depending on the duration of radiotherapy. This regimen is administered alongside external beam radiotherapy (EBRT) followed by brachytherapy.

In the KEYNOTE A18 study, EBRT was recommended with 23-28 fractions (1.8 Gy to 2 Gy per fraction) in total dose of 45 Gy to 50 Gy ± boost doses, followed by brachytherapy with 4-6 fractions (5 Gy to 7 Gy per fraction) in total dose of 25 Gy to 30 Gy.
