# Supplementary material for: The Polish Society of Gynecological Oncology Guidelines for the Diagnosis and Treatment of Cervical Cancer (v2024.0)
Source: J Clin Med. 2024 Jul 25;13(15):4351. doi: 10.3390/jcm13154351 (PMC11313441; doi:10.3390/jcm13154351)
Supplement: Supplementary file 1 [file jcm-13-04351-s001.zip › PSGO, File S6.pdf]

File S6: Systemic treatment.

Recommended therapy regimens for the treatment of persistence, metastatic or recurrence of cervical cancer.

Line of treatment	Drugs	Drug dosage	Rhythm of administering drugs	Number of cycles	References
First line therapy (PD-L1–positive tumors, CPS of ≥1)	Cisplatin	50 mg/m^2 IV infusion over 1 hour (day 1)	Every 21 days	6	[100]- IIA
	or carboplatin* instead cisplatin	AUC 5 IV infusion over 1 hour (day 1)			
	Paclitaxel	175 mg/m^2 infusion over 3 hours (day 1)	Every 21 days	6	
	Bevacizumab	15 mg/m^2 infusion over 90 to 30 minutes (day 1)	Every 21 days	up to PD or local practice[#]	
	Pembrolizumab	200 mg infusion over 1 hour (day 1)	Every 21 days	up to 35	
First line therapy	Cisplatin	50 mg/m^2 IV infusion over 1 hour (day 1)	Every 21 days	up to 6 cycles (if CR) or until PD	[103]- IIA
	or carboplatin* instead cisplatin	AUC 5 IV infusion over 1 hour (day 1)			
	Paclitaxel	175 mg/m^2 infusion over 3 hours (day 1)	Every 21 days	up to 6 cycles (if CR) or until PD	
	Bevacizumab	15 mg/m^2 infusion over 90 to 30 minutes (day 1)	Every 21 days	Patients who achieve a complete response after ≥6 treatment cycles may be allowed to continue only on bevacizumab plus atezolizumab	

	Atezolizumab	1200 mg infusion over 1 hour (day 1)	Every 21 days	Patients who achieve a complete response after ≥6 treatment cycles may be allowed to continue only on bevacizumab plus atezolizumab	
First line therapy	Cisplatin	50 mg/m² IV infusion over 1 hour (day 1)	Every 21 days	up to PD	[97]-IIA
	Paclitaxel	175 mg/m² infusion over 3 hours (day 1)	Every 21 days		
	Bevacizumab	15 mg/m² infusion over 90 to 30 minutes (day 1)	Every 21 days		
First line therapy†	Topotecan	0.75 mg/m² IV infusion over 30 minutes (day 1-3)	Every 21 days	up to PD	[97]-IIA
	Paclitaxel	175 mg/m² infusion over 3 hours (day 1)	Every 21 days		
	Bevacizumab	15 mg/m² infusion over 90 to 30 minutes (day 1)	Every 21 days		
Second- line or subsequent therapy	Tisotumab vedotin	2 mg/kg (up to a maximum of 200 mg for patients ≥100 kg) infusion over 30 minutes (day 1)	Every 21 days	up to PD	[106]-IIA
Second- line or subsequent therapy	Cemiplimab	350 mg infusion over 30 minutes (day 1)	Every 21 days	up to 96 weeks	[105]-IIA
Second- line or subsequent therapy	Vinorelbine	30 mg/m² IV infusion over 10 minutes (days 1 and 8)	Every 21 days	up to PD	[105]-IIA
Second- line or subsequent therapy	Gemcitabine	1000 mg/m² IV infusion over 30 minutes (days 1 and 8)	Every 21 days	up to PD	[105]-IIA

* Recommended as an alternative to cisplatin for patients who have previously received cisplatin with radiotherapy or impairment of renal function

There was no maximum number of bevacizumab administrations in KEYNOTE 826 study

† recommended as an alternative to cisplatin for patients who have impairment of renal function;

AUC: Area Under the Curve; PD-L1: Programmed Death-Ligand 1; CPS: Combined Positive Score; PD: Progressive Disease
